# Computational Analysis of the Asymmetric Hydrogenation of γ-Ketoacids: Weak Interactions and Kinetics

**DOI:** 10.3390/molecules31020385

**Published:** 2026-01-22

**Authors:** Ivan S. Golovanov, Evgeny V. Pospelov

**Affiliations:** N. D. Zelinsky Institute of Organic Chemistry, Russian Academy of Sciences, Leninsky Prospect 47, 119991 Moscow, Russia

**Keywords:** asymmetric catalysis, non-bonding interactions, asymmetric hydrogenation, transition metal catalysis, DFT calculations, theoretical investigations into asymmetric catalysis and synthesis

## Abstract

A computational study of the mechanism of asymmetric hydrogenation of γ-keto acids with the Ni(S,S)-QuinoxP* system was conducted. The main steps of the reaction mechanism were determined, including the formation of the NiH(S,S-QuinoxP*)^+^ complex starting from a γ-keto acid molecule and the involvement of the hydrogen “metathesis” step. The rate-limiting and stereo-determining step of the reaction was identified as the transfer of a hydrogen atom from the catalytic particle to the carbonyl group of the substrate molecule. The stereochemical outcome of the process was calculated. The influence of weak interactions on the stereoselectivity of the process was demonstrated using NCI and sobEDAw analyses.

## 1. Introduction

Asymmetric hydrogenation catalyzed by transition metal complexes (e.g., Ni, Cu, Fe, and Co) [1,2,3,4,5,6] is a rapidly developing area of modern chemistry. The possibility of replacing catalytic systems based on noble metals (Au, Ru, Rh, Ir, Pt, Pd) with relatively inexpensive and readily available systems based on transition metals opens a way to an inexpensive and highly stereoselective synthesis of various compounds, many of which are intermediates in the synthesis of pharmaceutically active compounds and natural products [7,8,9]. The synthesis of 5- and 6-membered lactones using γ/δ-keto acids, readily available from natural raw materials, has been actively studied in recent years [10]. Asymmetric hydrogenation of keto acids uses chiral catalysts (often Ir, Ru, Ni, or Co with specific ligands) to convert keto-functionalized acids into valuable chiral hydroxy acids or lactones, producing single enantiomers (optical isomers) crucial for pharmaceuticals, with high efficiency (TONs up to 100,000) and enantioselectivity (up to 99% ee) for γ- and δ-ketoacids, offering a green, scalable route to chiral building blocks and certain drugs (for example, Ezetimibe [11]). Catalytic systems based on Ir [11], Ru [12], and Pd [13] are used for synthesizing chiral lactones, as well as the recently proposed methods utilizing chiral Ni complexes [14,15]. Despite the availability of some experimental material, this topic has not yet been thoroughly studied. Moreover, this process lacks a sufficient explanation of its stereoselectivity and proposed mechanism. In this work, the mechanism of the Ni-catalyzed asymmetric hydrogenation reaction of γ-keto acids was studied using DFT calculations. In addition, the weak interactions contributing to the stereoselectivity of the studied reaction were visualized and quantified using NCI [16] and sobEDAw [17] analyses.

## 2. Results and Discussion

It can be noted that asymmetric hydrogenation reactions typically follow a specific pattern of elementary steps. The first step of any catalytic process is the formation of a catalytic particle. Considering that the starting compounds for the formation of the catalytic particle are nickel perchlorate and (S,S)-QuinoxP*, as well as the presence of hydrogen dissolved in the reaction mixture, it can be assumed that a number of nickel hydride complexes (including, but not limited to, mono- and dihydride complexes and their dimers) are formed during the reaction and can act as catalytic particles for the asymmetric hydrogenation process under consideration. Since in this case the catalytic system is of a “cocktail” type due to the possibility of interconversion of nickel hydride complexes into each other under these conditions, it is necessary to select a catalytic particle that, firstly, can exist under the reaction conditions, and secondly, effectively binds to the substrate molecules, namely a γ-keto acid. Thus, we decided to choose the hydride complex NiH(S,S-QuinoxP*)^+^ **1**, whose possible formation was demonstrated in one of our recent studies, as the catalytic particle [18].

The next step of the process is the interaction between the catalytic particle and the substrate molecule, leading to the formation of a prereaction complex. Note that although this stage itself is usually neither stereodetermining nor rate-limiting, the quality of the interaction between the catalytic particle and the substrate molecule at this step can significantly affect the subsequent steps. After the formation of the prereaction complex, hydride transfer occurs from the metal atom to the functional group of the substrate molecule. This step is often both rate-limiting and stereodetermining, since the hydrogen atom transferred to the substrate molecule typically does not participate further in the process, as shown by isotope labeling experiments in recent studies [1]. It is worth noting that the nature of the interactions between the substrate and catalytic particle molecules at this stage is of particular interest, as it can shed light on the possible influence of the interaction between the substrate and the catalyst (or part of it) on the stereoselectivity of the process.

Finally, a crucial step is the so-called hydrogen “metathesis,” without which the regeneration of the catalytic particle cannot occur. This step typically occurs with a high reverse barrier but a low forward barrier, suggesting that this step is irreversible within the energy profile of the transformation under consideration. However, hydrogen “metathesis” requires the hydrogen molecule to successfully coordinate with the metal atom, which is sometimes difficult because the ligand environment creates certain steric hindrance.

However, the ligand structure and the steric hindrance it causes could be a double-edged sword. On the one hand, the typically “branched” ligand structure complicates the interaction of the catalytic particle with substrate molecules, solvent, or hydrogen. On the other hand, it is the ligand skeleton that is responsible for the majority of the weak interactions that influence the stereochemical outcome of asymmetric hydrogenation reactions. Therefore, it is important to maintain a balance between hindering the kinetics of the process and improving the interaction between the catalytic particle and the substrate molecule.

**Scheme 1:**
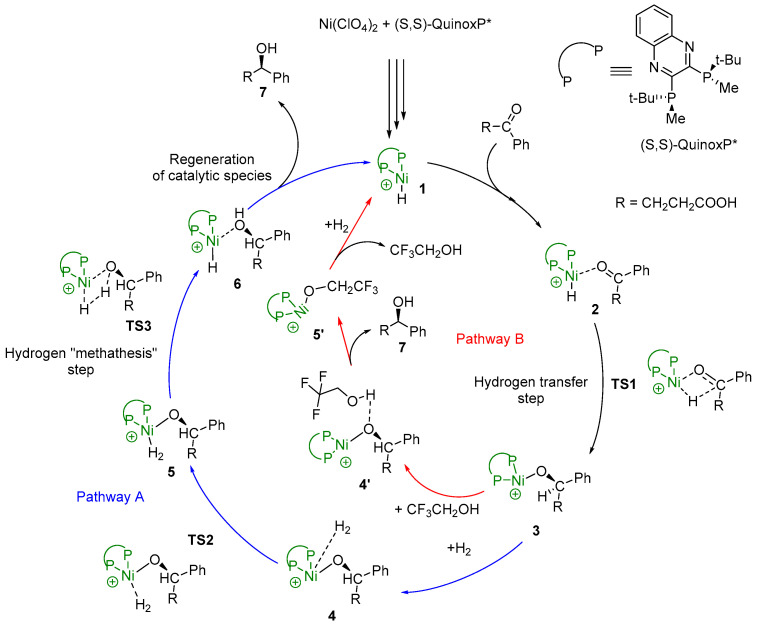
Possible catalytic cycle of the Ni-catalyzed asymmetric hydrogenation of γ-keto acids.

A reaction mechanism was proposed for the Ni-catalyzed asymmetric hydrogenation of γ-keto acids previously studied experimentally [14,15]. At the first step, complex **2** is formed from Ni catalyst **1** and a substrate molecule. The use of Ni-H species **1** as a catalyst has been substantiated in a number of previous works [1,6]. Then, the intramolecular addition of a H atom to the carbonyl carbon (hydrogen transfer step) occurs via **TS1** to give complex **3**. The process can proceed further through two pathways (pathway A and pathway B). Pathway A takes hydrogen, needed for product recovery and catalytic species regeneration, directly from the hydrogen molecule. Alternatively, pathway B utilizes a solvent, trifluoroethanol, as the hydrogen source, resulting in the formation of nickel alkoxide. In pathway A, at the next stage complex **4** is formed by the reaction of **3** with a hydrogen molecule (with a distance of 3.38 angstroms (pathway R) or 2.76 angstroms (pathway S) between the Ni atom and the hydrogen molecule). The subsequent “wedge-in” of the coordinated hydrogen molecule via **TS2** leads to species **5**. Finally, the “metathesis” stage of the hydrogen molecule in complex **5** via **TS3** results in particle **6**, which is actually a complex of the original catalytic particle and the γ-hydroxy acid. Their subsequent splitting completes the catalytic cycle and leads to the regeneration of the catalytic particle **1** (Scheme 1). The alternative pathway B involves the interaction of complex **3** with a trifluoromethanol molecule to give complex **4′**. After proton transfer from the alcohol molecule to the substrate molecule, the formation of the target hydroxy acid **7** and complex **5′** occurs. Then particle **5′** interacts with a hydrogen molecule, which leads to the formation of catalytic particle **1** and the closure of the catalytic cycle. Despite the fundamental possibility of the reaction occurring through either Pathway A or Pathway B, only Pathway A was calculated in this study, since previous studies [1] have demonstrated the possibility of hydrogen “metathesis” and shown that Pathway B requires overcoming a high activation barrier. Furthermore, regeneration of catalytic particle **1** still requires the interaction of **5′** with molecular hydrogen.

**Scheme 2:**
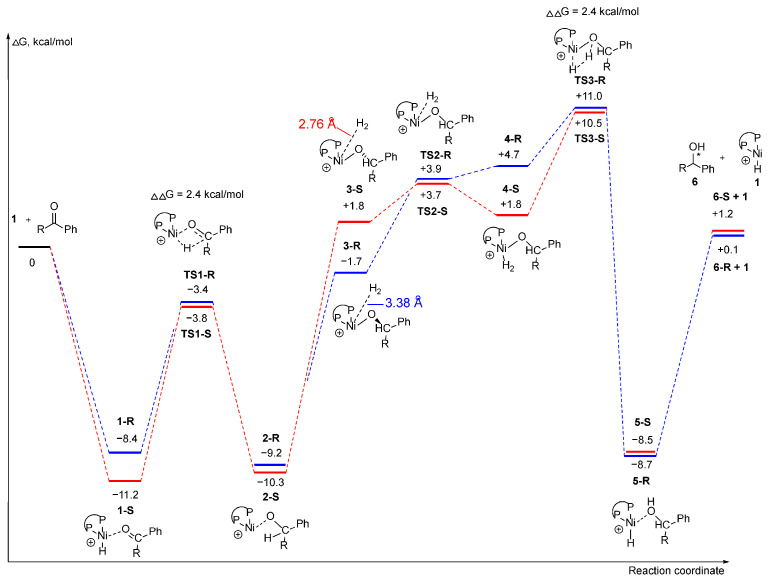
Computed Gibbs free energy profiles for the asymmetric hydrogenation of γ-ketoacids catalyzed by the Ni complex of (S,S)-QuinoxP*. Calculations were performed at DFT level of theory with TPSSh functional, using the def2-TZVPP basis set on Ni and def2-SVP on other atoms, in 2,2,2-trifluoroethanol (SMD model).

We optimized both singlet and triplet states of Ni complex **1** and discovered that triplet-singlet gap ∆E_T-S_ = +10.9 kcal/mol. Furthermore, for all other calculated structures, stability analysis indicates a stable wavefunction, so the ground state is the singlet state. Thus we used multiplicity *1* throughout calculations. Calculated Gibbs free energy profile of the Ni-catalyzed asymmetric hydrogenation of a γ-keto acid (the “blue” pathway for R and the “red” pathway for S) is shown in Scheme 2. In fact, the formation of pre-reaction complexes **1-R** and **1-S** is an energetically favorable process (with a benefit of 8.4 kcal/mol for **1-R** and 11.2 kcal/mol for **1-S**). Their conversion into alcoholates **2-S** and **2-R** occurs via transition states **TS1-R** and **TS1-S** with similar energies, which are discussed in more detail below. The subsequent interaction with a hydrogen molecule leads (without a transition state) to complexes **3-R** and **3-S**, with complex **3-R** being 3.5 kcal/mol more favorable than the corresponding complex **3-S**. The distance between the hydrogen molecule and the nickel atom in structures **3-R** and **3-S** deserves special attention. Indeed, the difference in the corresponding distances in **3-R** and **3-S** is almost 0.5 angstroms (2.76 angstroms in **3-S** and 3.28 angstroms in **3-R**). This explains the difference in the activation barriers at the step of incorporation of a hydrogen molecule into the inner coordination sphere of the nickel atom (**TS2-R** and **TS2-S**). The subsequent step of hydrogen “metathesis” occurs via transition states with similar energies **TS3-R** (ΔG = 11.0 kcal/mol) and **TS3-S** (ΔG = 10.5 kcal/mol) and with a high barrier to the reverse transformation (19.7 kcal/mol for **TS3-R** and 19.0 kcal/mol for **TS3-S**). Then, everything ends with the regeneration of catalytic species **1** and the formation of γ-hydroxycarboxylic acids **6-S** and **6-R**.

**Scheme 3:**
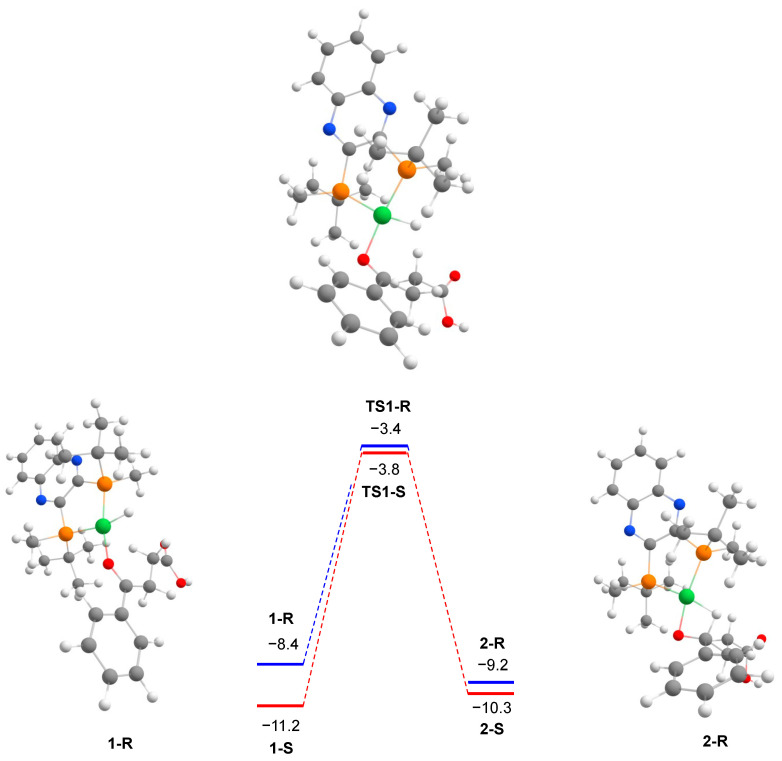
The stereodetermining step of the reaction. The structures of the R-pathway are shown. The structures of the minor S-pathway are omitted for clarity.

As shown in the energy diagram, the main difference in the Gibbs free energy of activation is observed at the first step of the process, i.e., transfer of the hydrogen atom from the Ni atom to the C atom of the carbonyl group, with a barrier of 5.0 kcal/mol for **TS1-R** and 7.4 kcal/mol for **TS1-S**, ΔΔG_act_ = 2.4 kcal/mol (Scheme 3).

To verify the reproducibility of the obtained result, this stage was recalculated using a different, suitable DFT functional, M11-L, yielded similar reaction barriers (4.5 kcal/mol for **TS1-R** and 7.2 kcal/mol for **TS1-S** (ΔΔG_act_ = 2.7 kcal/mol)).

The barrier of the “metathesis” of the hydrogen molecule is 6.3 kcal/mol for **TS3-R** and 8.7 kcal/mol for **TS3-S** (ΔΔG_act_ = 2.4 kcal/mol). Note that the barrier of the reverse reaction for the S-stereoisomer in step 1 is lower than that of the forward reaction. This should further promote the formation of the R-product.

To clarify the difference in the rates of the process under study for paths R and S, Eyring–Polanyi equation was used:

$$k\left(T\right)=\kappa \frac{k_{B}T}{h}e^{-∆G_{0}^{\neq }/RT}$$

k(T)—reaction rate constant; κ—transmission coefficient; $k_{B}$—Boltzmann constant; T—temperature; h—Planck constant; $∆G_{0}^{\neq }$—Gibbs free energy of activation; R—universal gas constant. Assuming that for the transformation of **1** into **2** via **TS1** the difference in transmission coefficients can be neglected, we obtain that the ratio k(R)/k(S) = 44.7.

**Figure 1:**
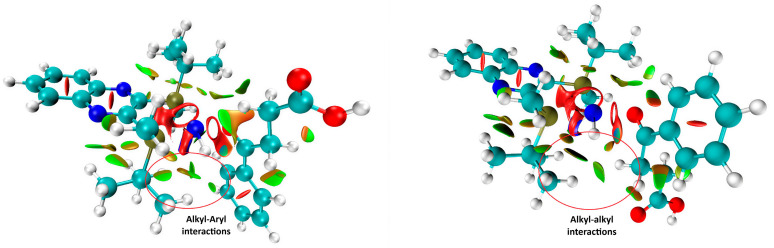
NCI analysis for **TS1-R** (**left**) and **TS1-S** (**right**).

Considering that the addition of the hydrogen atom, which determines the stereochemistry of the process, occurs at the **1** ⟶ **2** conversion step via **TS1**, NCI analysis was performed for **TS1-R** and **TS1-S** to visualize the weak interactions between the substrate molecule and the catalyst particle (Figure 1, see Supplementary Materials for color code for NCI).

NCI analysis reveals that in the case of **TS1-R**, interactions are observed between the π-orbitals of the phenyl group of the substrate and the tert-butyl group of the catalyst, leading to the stabilization of the transition state. Although such interactions are not observed in the case of **TS1-S**, they are replaced by interactions between the CH bonds of the substrate molecule and the tert-butyl moiety of the catalyst, which also leads to enhanced interactions between the substrate and the catalyst particle. To elucidate the nature of these interactions in **TS1-R** and **TS1-S** and to quantify them, energy decomposition analysis (sobEDAw) was also performed (Table 1).

Apparently, the main difference between **TS1-R** and **TS1-S** lies in the dispersion and electrostatic interactions. The electrostatic energy and exchange-repulsion energy were found to be higher in **TS1-R** than in **TS1-S**, while the dispersion interaction in **TS1-R** is significantly lower (by almost 3 kcal/mol) than in **TS1-S**. In summary, **TS1-R** has a lower interaction energy than **TS1-S**, which is consistent with experiments [14,15]. The large difference in dispersion energy may indicate that the ligand skeleton and the apparently non-reactive moiety of the substrate molecule may interact with each other and thereby improve the enantioselectivity of the reaction.

**Figure 2:**
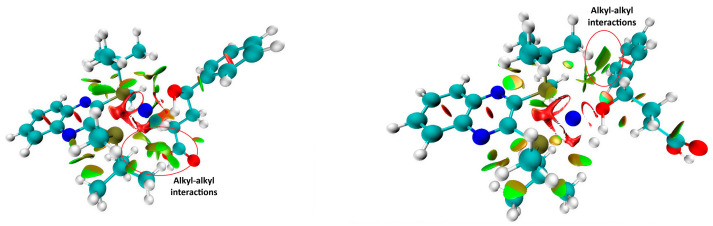
NCI analysis for **TS3-R** (**left**) and **TS3-S** (**right**).

The kinetics of the “metathesis” step also predicts the preferential formation of the R-isomer of the γ-hydroxy acid (see discussion above). The formation of the R-isomer was also supported by NCI (Figure 2) and sobEDAw analyses of the corresponding transition states **TS3-R** and **TS3-S**.

According to NCI for **TS3-R**, a strong interaction exists between the alkyl tail of the substrate molecule and the tert-butyl group of the ligand moiety. Furthermore, an interaction is also observed between the alpha-aromatic hydrogen atom of the substrate molecule and the methyl group of the catalytic particle backbone. It is worth noting that such interactions are almost nonexistent in **TS3-S**. In turn, **TS3-S** exhibits the interaction between the tert-butyl group of the ligand and the alpha-aromatic hydrogen atom of the substrate molecule, as well as the weak interaction between the hydrogen atoms of the phenyl group and the methyl group of the ligand. No other weak interactions leading to improved binding between the substrate and catalyst molecules are observed.

To quantify the different components of the binding energy between the substrate and catalyst molecules in **TS3-R** and **TS3-S**, energy decomposition analysis (sobEDAw) was also performed (Table 2).

Overall, the interaction between the molecules of the catalytic particle and the substrate is stronger in **TS3-R** than in **TS3-S**. Thus, the difference in the total interaction energy between **TS3-R** and **TS3-S** is 4.37 kcal/mol. The largest difference in its constituent energies lies in the electrostatic interaction (12.33 kcal/mol), as well as in the orbital (8.29 kcal/mol) and dispersion (8.34 kcal/mol) interactions. We suggest that the main contribution to the latter interactions is made by the weak interactions between the ligand skeleton and various moieties of the initial γ-keto acid molecule. It is worth mentioning separately that the exchange-repulsion energy is also higher in **TS3-R**; however, the total contribution of all components still leads to better binding of the substrate and catalyst molecules than in **TS3-S**.

**Figure 3:**
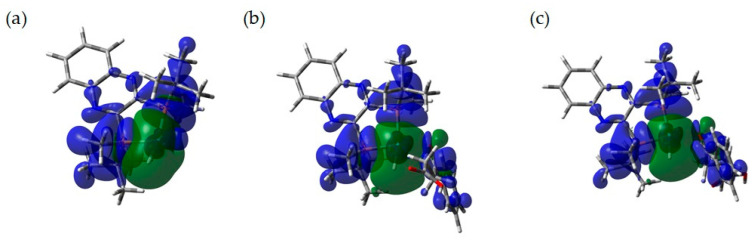
(**a**) Visualization of NOCV pair density isosurfaces for **1**. (**b**) Visualization of NOCV pair density isosurfaces for **TS1-R**. (**c**) Visualization of NOCV pair density isosurfaces for **TS1-S**. The blue/green colored isodensity surface defines electron density depletion/accumulation.

To get insights into the electron distribution in catalytic species **1** and transition states of hydrogen transfer step (**TS1**), we examined them using the Extended Transition State—Natural Orbitals for Chemical Valence method (ETS-NOCV). In **1** the main part of contribution to ΔEorb stems from the sum of the first and second NOCV pairs (ΔE = −101.2 kcal/mol). As follows from the visualization of NOCV pair density isosurface, it is clear that the sum of two first NOCV pairs corresponds to electron donation from both phosphorus and nitrogen atoms of the ligand to the nickel and hydrogen atoms (Figure 3).

In **TS1** unlike in **1**, the π orbitals of the aromatic moiety of the substrate molecule also participate in the electron distribution in the transition state. This may indicate the influence of the substrate’s structural features on the reaction and may indirectly explain the different stereochemical outcomes of substrates that differ only in the substitution pattern of the aromatic moiety. It should be noted that the first two NOCV pairs also make a significant contribution to ΔE_orb_ (ΔE = −116.8 kcal/mol for **TS1-R** and ΔE = −115.9 kcal/mol for **TS1-S**).

## 3. Materials and Methods

DFT calculations were performed using Gaussian 16 Rev.C01 [19]. The TPSSh DFT functional with the def2-TZVPP basis set on Ni and def2-SVP on the other atoms was used for geometry optimization, thermodynamic and kinetic calculations. All calculations were performed in 2,2,2-trifluoroethanol (SMD model). Noncovalent interaction (NCI) analysis [16] and ETS-NOCV [20] were performed using Multiwfn ver. 3.8. [21], while sobEDAw analysis was performed according to the reported procedure [17]. See Supplementary Materials for details.

## 4. Conclusions

A mechanism for the asymmetric hydrogenation of γ-keto acids was proposed and modeled by DFT calculations. The stereoselectivity of the process was shown to be influenced by both the reaction kinetics and weak interactions in the transition states of the stereodetermining step–hydrogen atom transfer stage. DFT computations have shown that the difference in the activation barriers of this stage is 2.4 kcal/mol, which determines the stereoselectivity of the formation of the R-product. The difference in Gibbs free energy between the R and S pathways predicts 96% ee for the hydrogenation product, in good agreement with the experimental data [14,15]. Using the Eyring–Polanyi equation, the ratio of the rate constants for pathways R and S can be estimated as k(R)/k(S) = 44.7. According to NCI and sobEDAw analyses, weak interactions in **TS1** between π-orbitals of the phenyl group of the substrate and the tert-butyl group of the catalyst have an impact on preferential formation of the R-product.

## Figures and Tables

**Table 1:** SobEDAw analysis for **TS1-R** and **TS1-S**.

E	TS1-R, kcal/mol	TS1-S, kcal/mol
E_total_	−38.40	−37.64
E_els_	−46.62	−47.89
E_xrep_	67.74	66.88
E_orb_	−35.91	−35.72
E_disp_	−23.62	−20.92

**Table 2:** SobEDAw analysis for **TS3-R** and **TS3-S**.

E	TS3-R, kcal/mol	TS3-S, kcal/mol
E_total_	−254.70	−250.33
E_els_	−273.02	−260.69
E_xrep_	217.82	193.24
E_orb_	−163.49	−155.20
E_disp_	−36.02	−27.68

## Data Availability

All data are included in the manuscript and the Supplementary Materials.

## Supplementary Materials

The following supporting information can be downloaded at: https://www.mdpi.com/article/10.3390/molecules31020385/s1, Details of DFT calculations, Cartesian coordinates and energies for all calculated structures.
