# Supplementary material for: Computational Analysis of the Asymmetric Hydrogenation of γ-Ketoacids: Weak Interactions and Kinetics
Source: Molecules. 2026 Jan 22;31(2):385. doi: 10.3390/molecules31020385 (PMC12844336; doi:10.3390/molecules31020385)
Supplement: Supplementary file 1 [file molecules-31-00385-s001.zip › molecules-4021365-supplementary.pdf]

Supporting Information

Computational analysis of the asymmetric hydrogenation of γ-ketoacids: weak interactions and kinetics

Ivan S. Golovanov[1],* and Evgeny V. Pospelov[1],*

1 N. D. Zelinsky Institute of Organic Chemistry, Russian Academy of Sciences, Leninsky Prospect, 47, 119991, Moscow, Russian Federation; igolovanov@ioc.ac.ru; evpospelov@ioc.ac.ru.

Table of contents

1. DFT calculations.. S3
1.1. General information.. S3
1.2. Cartesian coordinates and energies.. S4
1.3. NCI analysis S59
1.4. SobEDAw analysis..S64
1.5. ETS-NOCV analysis..S66

1. DFT calculations

1.1. General information

DFT calculations were performed with Gaussian 16 Rev.C01. TPSSh DFT functional with def2-TZVPP basis set on Ni and def2-SVP on the other atoms was used for geometry optimization, calculations of thermodynamics and kinetics. Calculations were performed in 2,2,2-trifluoroethanol (SMD model). All calculations were done at 318.15K. Cartesian coordinates are given in angstroms; absolute energies for all substances are given in hartrees. Analysis of vibrational frequencies was performed for all optimized structures. All compounds, except transition state structures, were characterized by only real vibrational frequencies. TS were characterized by one imaginary frequency. Wavefunction stability, using *stable* keyword, was also checked for each molecule.

For calculations of optimized geometries, frequencies and thermodynamics, following keywords were used:

# opt freq tpssh gen scrf=(smd,solvent=2,2,2-trifluoroethanol) scf=qc nosymm temperature=318.15 test

The same parameters were calculated for transition state structures with keywords:

# opt=(calcfc,ts,noeigentest) freq tpssh gen scrf=(smd,solvent=2,2,2-trifluoroethanol) nosymm scf=qc temperature=318.15 test

IRC calculation was performed for TS and proved that TS connects products and reactants:

# irc=(forward,calcfc,maxcycle=150,MaxPoints=5,ReCorrect=Never,HPC) tpssh scf=qc gen scrf=(smd,solvent=2,2,2-trifluoroethanol) nosymm temperature=318.15 test

# irc=(reverse,calcfc,maxcycle=150,MaxPoints=5,ReCorrect=Never,HPC) tpssh scf=qc gen scrf=(smd,solvent=2,2,2-trifluoroethanol) nosymm temperature=318.15 test

We have also calculated key reaction step using M11-L DFT functional. Keyword *tpssh* was changed to *M11L*, other keywords remained the same. Cartesian coordinates and energies for m11L calculations are located separately at the end of 1.2 section of SI.

Reaction rate constant was calculated by the formula:

$$k(T) = \kappa \frac{k_B T}{h} e^{-\Delta G_0^{\neq}/RT}$$

$k(T)$ – reaction rate constant; κ – transmission coefficient; k_B – Boltzmann constant; T – temperature; h – Planck constant; $\Delta G_0^{\neq}$ – Gibbs free energy of activation; R – universal gas constant.

1.2. Cartesian coordinates and energies

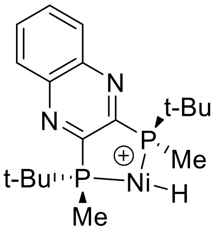

1

Charge 1; multiplicity 1

Ni	0.60534300	-0.64616100	0.26570300
P	-0.98056800	-1.44280700	1.35132500
P	-0.71303900	0.37807800	-1.21334800
C	-2.52555200	-0.66363300	0.66644300
C	-2.41428100	0.09691000	-0.54337300
H	1.24592500	-1.34332600	1.41961400
N	-3.67890100	-0.83412200	1.28006500
N	-3.47814900	0.59753800	-1.14280600
C	-4.78814400	-0.29623500	0.70114900
C	-6.05638300	-0.44661900	1.32166100
H	-6.11228500	-0.98098300	2.27217700
C	-4.68830100	0.40904900	-0.54739400
C	-5.86363100	0.92783900	-1.15252500
C	-7.18067900	0.07406100	0.71197700
C	-7.08446000	0.75979900	-0.52954200
H	-5.77068000	1.45680400	-2.10319900
H	-7.99081000	1.15917300	-0.99063000
H	-8.15925700	-0.04189000	1.18372700
C	-1.18487900	-3.30689900	1.10788600
C	-2.37747900	-3.82239200	1.93217500
C	-1.41270800	-3.55545400	-0.39280000
C	0.10825200	-4.00066400	1.56916300
H	-2.20648900	-3.70972000	3.01376900
H	-3.31574100	-3.31202900	1.67047800
H	-2.50327300	-4.89833300	1.72320800
H	-0.57339700	-3.17143400	-0.99542600
H	-1.48827000	-4.64139200	-0.56952200
H	-2.34524200	-3.09118000	-0.75182000
H	-0.03341000	-5.09177000	1.49015100
H	0.96766800	-3.72040800	0.94152000
H	0.35070200	-3.76884000	2.61853700
C	-1.07116000	-1.08382600	3.13837500
H	-2.08080300	-1.30829700	3.51188300
H	-0.32264700	-1.68775400	3.67092900
H	-0.85161500	-0.01689100	3.29104300
C	-0.43610400	2.24647200	-1.22601100
C	-1.34303200	2.97365700	-2.22746500
C	-0.68491500	2.75289900	0.20515300
C	1.04585600	2.43943900	-1.60138100
H	-1.13858400	2.66469600	-3.26433600

H	-2.40811900	2.79935600	-2.01473700
H	-1.15194600	4.05870300	-2.15924700
H	-0.07084000	2.20518200	0.94019300
H	-0.41455400	3.82060700	0.26576700
H	-1.74299000	2.65613800	0.49631700
H	1.28329000	3.51682500	-1.60215300
H	1.71416800	1.94553800	-0.87572500
H	1.27092900	2.04567500	-2.60560300
C	-0.84001100	-0.17095800	-2.95772200
H	-1.69621700	0.30858700	-3.45445800
H	0.09071700	0.08140500	-3.48757300
H	-0.97333000	-1.26292000	-2.97068900

DFT TPSSh; def2-TZVPP basis set on Ni, def2-SVP on the other atoms; 2,2,2-trifluoroethanol, SMD model
Total electronic energy= -3003.200575 E_0
Sum of electronic and zero-point Energies= -3002.768606 $E_0 + E_{ZPE}$
Sum of electronic and thermal Energies= -3002.737735 $E_0 + E_{tot}$
Sum of electronic and thermal Enthalpies= -3002.736728 $E_0 + H_{corr}$
Sum of electronic and thermal Free Energies= -3002.830026 $E_0 + G_{corr}$
Zero-point correction (*unscaled*) = 0.431969
Number of imaginary vibrational frequencies = 0

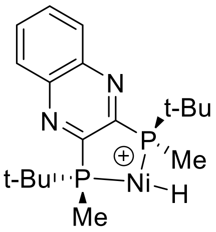

1

Charge 1; multiplicity **3**

Ni	0.78363500	-0.69418700	0.12512900
P	-1.01573300	-1.43406300	1.41823100
P	-0.74820000	0.34673800	-1.30160700
C	-2.52769100	-0.65238700	0.68458100
C	-2.42044800	0.06089500	-0.55750900
H	2.02990100	-1.21702700	0.90759800
N	-3.68469600	-0.78375400	1.30678900
N	-3.48720800	0.54015100	-1.17037400
C	-4.79272200	-0.26397700	0.71253500
C	-6.05915300	-0.38087700	1.34468000
H	-6.11294100	-0.87153300	2.31856400
C	-4.69623700	0.38307300	-0.56670100
C	-5.87167300	0.87707700	-1.19225400
C	-7.18382300	0.11463300	0.71528700
C	-7.09035700	0.74163700	-0.55751100
H	-5.78043200	1.36099800	-2.16676900
H	-7.99754800	1.12143300	-1.03326500
H	-8.16118700	0.02359100	1.19491100
C	-1.20985300	-3.29468300	1.15983100
C	-2.52276300	-3.83121100	1.74588300
C	-1.15111300	-3.52838500	-0.36050400
C	-0.00757100	-3.96930300	1.84667000
H	-2.58603500	-3.65940400	2.83165300
H	-3.40488600	-3.38034500	1.26848900
H	-2.56404300	-4.92102400	1.57585300
H	-0.21209900	-3.14330000	-0.79485700
H	-1.19335300	-4.61137600	-0.56502500
H	-1.99843000	-3.05438400	-0.88183600
H	-0.05265700	-5.05417900	1.65245000
H	0.95138000	-3.59241800	1.45786400
H	-0.02362700	-3.82337700	2.93801900
C	-1.15572600	-1.06767200	3.20285300
H	-2.12958400	-1.39967600	3.59162300
H	-0.34056600	-1.57205100	3.74148300
H	-1.05607100	0.01970800	3.34043900
C	-0.44231800	2.20657400	-1.22443400
C	-1.44240700	3.01346000	-2.06115900
C	-0.52007000	2.59415800	0.26256500
C	0.99179000	2.41754000	-1.74702100
H	-1.36910600	2.76719900	-3.13195700
H	-2.47972000	2.84709100	-1.73565400
H	-1.21584200	4.08783600	-1.94872800

H	0.16177900	1.97710700	0.87569300
H	-0.21829500	3.64799900	0.38338900
H	-1.54075100	2.48634700	0.66319300
H	1.24648000	3.48849100	-1.67716000
H	1.72739400	1.85290700	-1.14915600
H	1.09444000	2.11543800	-2.80134800
C	-0.96874000	-0.12976700	-3.05408900
H	-1.85071600	0.37044300	-3.48033600
H	-0.06689200	0.14588700	-3.62045200
H	-1.10353600	-1.22056000	-3.10564400

DFT TPSSh; def2-TZVPP basis set on Ni, def2-SVP on the other atoms; 2,2,2-trifluoroethanol, SMD model
Total electronic energy= -3003.183224 E_0
Sum of electronic and zero-point Energies= -3002.752326 $E_0 + E_{ZPE}$
Sum of electronic and thermal Energies=-3002.721186 $E_0 + E_{tot}$
Sum of electronic and thermal Enthalpies= -3002.720178 $E_0 + H_{corr}$
Sum of electronic and thermal Free Energies= -3002.815082 $E_0 + G_{corr}$
Zero-point correction (*unscaled*) = 0.430898
Number of imaginary vibrational frequencies = 0

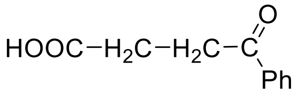

4-oxo-4-phenylbutanoic acid

Charge 0; multiplicity 1

C	3.53636300	0.05876200	-0.31203200
O	2.55633300	-0.67932400	-0.37448200
C	3.65258700	1.09550100	0.78783800
C	2.42548500	1.11238100	1.69809400
H	3.81330600	2.08449900	0.32595400
H	4.56638800	0.88094400	1.36710000
H	1.51391400	1.36011800	1.13824000
H	2.27688900	0.10886600	2.13347100
C	2.52708900	2.08116000	2.84876800
O	1.63172000	2.82092700	3.21510500
O	3.71699000	2.03148100	3.47140100
H	3.69994600	2.66499100	4.21815300
C	4.63607600	-0.05701000	-1.31901500
C	5.78966700	0.74954500	-1.28052300
C	4.50234800	-1.01743000	-2.34075800
C	6.78750900	0.59529400	-2.24709200
H	5.91695200	1.49939400	-0.49746300
C	5.49866300	-1.16883600	-3.30475300
H	3.60396400	-1.63837400	-2.36071900
C	6.64384800	-0.36144400	-3.25906400
H	7.68040400	1.22419700	-2.21076700
H	5.38623600	-1.91590100	-4.09450600
H	7.42575500	-0.47895700	-4.01386900

DFT TPSSh; def2-TZVPP basis set on Ni, def2-SVP on the other atoms; 2,2,2-trifluoroethanol, SMD model
Total electronic energy= -612.38345 E_0
Sum of electronic and zero-point Energies= -612.202337 $E_0 + E_{ZPE}$
Sum of electronic and thermal Energies= -612.189028 $E_0 + E_{tot}$
Sum of electronic and thermal Enthalpies= -612.188020 $E_0 + H_{corr}$
Sum of electronic and thermal Free Energies= -612.246424 $E_0 + G_{corr}$
Zero-point correction (*unscaled*) = 0.181116
Number of imaginary vibrational frequencies = 0

1-R

Charge 1; multiplicity 1

Ni	0.71082600	-0.76081400	0.17912500
C	3.50141900	0.11798000	-0.36795600
P	-0.91755400	-1.50734200	1.28710400
P	-0.62826600	0.43136300	-1.15914100
C	-2.46303400	-0.68714100	0.65756200
C	-2.34206800	0.10019000	-0.53382500
H	1.27252000	-1.71291200	1.17750200
N	-3.62145000	-0.85816100	1.26418400
N	-3.40257500	0.60505100	-1.13650600
C	-4.72484400	-0.30570200	0.68827400
C	-5.99647600	-0.45359300	1.30335200
H	-6.05823300	-0.99112700	2.25179500
C	-4.61761800	0.40620100	-0.55507200
C	-5.78972200	0.92654900	-1.16595600
C	-7.11699100	0.07109500	0.69032300
C	-7.01394600	0.75855400	-0.54982300
H	-5.69158300	1.45882700	-2.11432800
H	-7.91747400	1.16019500	-1.01455900
H	-8.09790300	-0.04378400	1.15755500
O	2.42250500	-0.45520000	-0.61140800
C	-1.25277700	-3.36584100	1.13087900
C	-2.41486200	-3.79033200	2.04522000
C	-1.59002600	-3.65073800	-0.34283500
C	0.02037100	-4.12979400	1.53388800
H	-2.16543200	-3.65799600	3.10947500
H	-3.33702800	-3.23175200	1.83098300
H	-2.61566500	-4.86267100	1.88021600
H	-0.78044500	-3.31788900	-1.01324900
H	-1.71805000	-4.73752300	-0.48096800
H	-2.52630200	-3.16092000	-0.65508200
H	-0.20087500	-5.21057400	1.51746200
H	0.85061500	-3.94023100	0.83735500
H	0.35332300	-3.87192200	2.55225300
C	-0.89848200	-1.09823200	3.07152400
H	-1.90672900	-1.20375900	3.49749400
H	-0.19773800	-1.77038800	3.58815100
H	-0.55126900	-0.06125700	3.19416800
C	-0.48302100	2.32229700	-1.20776400
C	-1.38158800	2.94039000	-2.29011000
C	-0.86378600	2.86431100	0.18069800
C	0.99160700	2.64378100	-1.50980200
H	-1.06995200	2.63750100	-3.30171700
H	-2.43750100	2.66730200	-2.15151200
H	-1.29794500	4.03955800	-2.23188300
H	-0.26829700	2.39894700	0.98352200
H	-0.67210900	3.95048900	0.20865600
H	-1.93077800	2.70786300	0.40472400
H	1.10532600	3.73557400	-1.62129000
H	1.64915600	2.31829300	-0.69158400

H	1.33644500	2.17440500	-2.44529800
C	-0.69650000	-0.12704800	-2.90729000
H	-1.57088900	0.29455500	-3.42414500
H	0.22739000	0.17900100	-3.42085800
H	-0.75981900	-1.22535500	-2.91794100
C	3.69090100	0.99701900	0.84681200
C	2.55864700	0.92386400	1.86402000
H	3.81048700	2.02700000	0.46562800
H	4.65365500	0.74467200	1.31681900
H	1.56935800	0.90590100	1.36764200
H	2.61918000	-0.00931000	2.44654900
C	2.49044800	2.04305500	2.87179600
O	1.49100500	2.29317400	3.52425600
O	3.63346500	2.72646000	3.00426900
H	3.50765500	3.41962100	3.68537900
C	4.60862800	-0.04613800	-1.33675200
C	5.85050900	0.60036000	-1.16602500
C	4.40870700	-0.86527000	-2.46890500
C	6.86579000	0.42971200	-2.10911800
H	6.03113100	1.23972800	-0.30083700
C	5.42674900	-1.03462100	-3.40500000
H	3.44701200	-1.36429100	-2.60153200
C	6.65743600	-0.38683200	-3.22709500
H	7.82417600	0.93523700	-1.97012100
H	5.26409800	-1.67192500	-4.27736300
H	7.45546900	-0.51941200	-3.96197800

DFT TPSSh; def2-TZVPP basis set on Ni, def2-SVP on the other atoms; 2,2,2-trifluoroethanol, SMD model
Total electronic energy= -3615.622688 E_0
Sum of electronic and zero-point Energies= -3615.007339 $E_0 + E_{ZPE}$
Sum of electronic and thermal Energies= -3614.961735 $E_0 + E_{tot}$
Sum of electronic and thermal Enthalpies= -3614.960727 $E_0 + H_{corr}$
Sum of electronic and thermal Free Energies= -3615.089854 $E_0 + G_{corr}$
Zero-point correction (*unscaled*) = 0.615349
Number of imaginary vibrational frequencies = 0

TS1-R

Charge 1; multiplicity 1

Ni	0.53177200	0.08674600	0.33637300
C	3.07591700	0.33239500	-0.04046300
P	-0.89453200	-1.15190600	1.27502300
P	-1.01042900	1.06604000	-0.94098600
C	-2.56270700	-0.71308700	0.57577600
C	-2.59625800	0.21323800	-0.51772100
H	1.30866700	-0.71290100	1.33355700
N	-3.66129600	-1.26212100	1.05517900
N	-3.71717500	0.48104800	-1.16171100
C	-4.83802600	-0.96848000	0.43574300
C	-6.05145300	-1.53094600	0.91352800
H	-6.01724700	-2.17439700	1.79509300
C	-4.85987600	-0.11128300	-0.71726800
C	-6.09141400	0.13858700	-1.37937000
C	-7.23474800	-1.26263700	0.25425700
C	-7.25405000	-0.42964000	-0.89765700
H	-6.08910100	0.78702000	-2.25796700
H	-8.20404800	-0.23735700	-1.40190000
H	-8.17023400	-1.69592700	0.61581000
O	2.07650600	0.94536800	-0.51088500
C	-0.67087300	-2.99723600	0.90434900
C	-1.75396800	-3.83544600	1.60492500
C	-0.75489200	-3.16919400	-0.62209500
C	0.71553200	-3.43457700	1.40792600
H	-1.67249400	-3.77353000	2.70121500
H	-2.76938000	-3.53268500	1.31306200
H	-1.61412500	-4.89191800	1.31919800
H	0.00241900	-2.55597200	-1.13804800
H	-0.56806000	-4.22549600	-0.87856700
H	-1.74878200	-2.89912800	-1.01397800
H	0.81669000	-4.52173400	1.24996600
H	1.52840200	-2.93406100	0.86140600
H	0.84542400	-3.23988400	2.48453300
C	-1.12111800	-0.97283000	3.08075500
H	-2.04688400	-1.47439500	3.39802000
H	-0.25567000	-1.40688900	3.60207100
H	-1.18450400	0.09878000	3.32066700
C	-1.33182700	2.90290800	-0.60262900
C	-2.38723300	3.48677300	-1.55348500
C	-1.79290700	3.02713500	0.85968100
C	0.00902900	3.63515400	-0.79511800
H	-2.04419400	3.47766400	-2.59969500
H	-3.34142400	2.94317900	-1.49428400
H	-2.57198700	4.53833000	-1.27320000
H	-1.06494400	2.57582000	1.55401900
H	-1.89065200	4.09468200	1.12000500
H	-2.77278000	2.55085700	1.02450500
H	-0.15078800	4.71618500	-0.64275100
H	0.76536400	3.29769000	-0.07112300

H	0.41284500	3.49671900	-1.81092700
C	-0.86891000	0.87555700	-2.75907500
H	-1.79263500	1.19303600	-3.26458100
H	-0.01994100	1.47599400	-3.11867300
H	-0.67678200	-0.18421100	-2.98336300
C	3.71380100	0.79165800	1.24841900
C	2.92252900	1.88304500	1.96121200
H	4.72203700	1.15639500	0.98333100
H	3.87499800	-0.07844300	1.90356200
H	2.84872000	2.77577000	1.31678500
H	1.89370900	1.55422900	2.17212400
C	3.52285900	2.33937400	3.26732300
O	2.87050000	2.65972800	4.24443200
O	4.86332600	2.39185500	3.24503900
H	5.17503600	2.71901200	4.11416100
C	3.71767400	-0.73085600	-0.84488000
C	4.86013300	-1.42441600	-0.39263900
C	3.17887400	-1.04097900	-2.11250000
C	5.44342500	-2.40925400	-1.19144800
H	5.29777200	-1.20131500	0.58149700
C	3.76740200	-2.02257900	-2.90697400
H	2.29852600	-0.49886800	-2.46279400
C	4.89977200	-2.70964000	-2.44662300
H	6.32659400	-2.94401100	-0.83461400
H	3.34668500	-2.25534500	-3.88797300
H	5.36037100	-3.48077000	-3.06926700

DFT TPSSh; def2-TZVPP basis set on Ni, def2-SVP on the other atoms; 2,2,2-trifluoroethanol, SMD model
Total electronic energy= -3615.613340 E_0
Sum of electronic and zero-point Energies= -3614.999214 $E_0 + E_{ZPE}$
Sum of electronic and thermal Energies= -3614.954033 $E_0 + E_{tot}$
Sum of electronic and thermal Enthalpies= -3614.953025 $E_0 + H_{corr}$
Sum of electronic and thermal Free Energies= -3615.081893 $E_0 + G_{corr}$
Zero-point correction (*unscaled*) = 0.614186
Number of imaginary vibrational frequencies = 1; 50i

2-R

Charge 1; multiplicity 1

Ni	0.78766900	0.16467700	-0.12001200
C	2.95190500	0.18504400	-0.11063200
P	-0.62971500	-1.08206300	0.97289100
P	-0.82041800	1.14751800	-1.18863700
C	-2.31634000	-0.64708000	0.35663600
C	-2.38754600	0.28847300	-0.72274100
H	2.19026200	-0.50689400	0.53876500
N	-3.39778200	-1.21001200	0.85987300
N	-3.52352800	0.56543500	-1.33230900
C	-4.59100400	-0.91354400	0.27589900
C	-5.78795000	-1.49018900	0.77773700
H	-5.72621600	-2.14689500	1.64783700
C	-4.65021300	-0.03837000	-0.86434300
C	-5.90060400	0.21447600	-1.48839300
C	-6.98986300	-1.21850000	0.15512200
C	-7.04573200	-0.36810200	-0.98309000
H	-5.92584900	0.87578300	-2.35681500
H	-8.01047600	-0.17430800	-1.45775400
H	-7.91264400	-1.66276300	0.53537500
O	2.20430600	1.04458200	-0.87378000
C	-0.42898800	-2.93615000	0.67051700
C	-1.40127800	-3.75286200	1.53608400
C	-0.68445500	-3.18839100	-0.82534400
C	1.02343800	-3.29548100	1.02703700
H	-1.18911700	-3.63568700	2.60999100
H	-2.44922600	-3.47513400	1.35330300
H	-1.27814800	-4.82050200	1.28578000
H	-0.02395000	-2.57338800	-1.45874100
H	-0.47913000	-4.24801800	-1.05181500
H	-1.72966900	-2.98157800	-1.10486300
H	1.16239100	-4.38222200	0.89950700
H	1.74084200	-2.78422300	0.36768000
H	1.26591800	-3.04759300	2.07283000
C	-0.71181600	-0.81006500	2.78041400
H	-1.57870600	-1.33474700	3.20811000
H	0.21608700	-1.17360400	3.24610900
H	-0.80942000	0.26964000	2.96594800
C	-1.07234600	2.96912600	-0.75032000
C	-2.23126100	3.57068400	-1.56247300
C	-1.37121200	3.04119000	0.75675700
C	0.23297100	3.71760400	-1.07230200
H	-2.01950700	3.56756400	-2.64269000
H	-3.17979000	3.04260200	-1.38970900
H	-2.36078900	4.62098400	-1.25013100
H	-0.55429400	2.60402800	1.35311800
H	-1.47627000	4.09884500	1.05081100
H	-2.30998800	2.52590200	1.01648600
H	0.08873800	4.78540100	-0.83678100
H	1.07776900	3.34550600	-0.47539800

H	0.49836700	3.64271400	-2.13844500
C	-0.70346800	0.99885000	-3.00520000
H	-1.62640900	1.36420500	-3.47898200
H	0.16032300	1.58039300	-3.35878000
H	-0.55321100	-0.06094300	-3.25754900
C	3.77912700	0.88565500	0.98746600
C	2.93015500	1.81393200	1.85837700
H	4.56769200	1.45734500	0.47324000
H	4.27447800	0.12533500	1.60982900
H	2.52280200	2.62977500	1.23867300
H	2.08106800	1.27270400	2.30219000
C	3.69470800	2.45358100	2.98880100
O	3.32671100	2.48244300	4.14948300
O	4.84196900	3.02109100	2.58180100
H	5.28130600	3.42400000	3.35896000
C	3.76049100	-0.83015600	-0.91512400
C	4.39400500	-1.91747900	-0.28858400
C	3.90404400	-0.65987900	-2.30019300
C	5.15552700	-2.81987100	-1.03793400
H	4.28921400	-2.06574500	0.78955400
C	4.67086600	-1.56102900	-3.04793100
H	3.41329300	0.18537600	-2.78683300
C	5.29732200	-2.64339700	-2.42008200
H	5.63930100	-3.66437200	-0.54028000
H	4.77976200	-1.41460700	-4.12586100
H	5.89434900	-3.34802900	-3.00481200

DFT TPSSh; def2-TZVPP basis set on Ni, def2-SVP on the other atoms; 2,2,2-trifluoroethanol, SMD model
Total electronic energy= -3615.627060 E_0
Sum of electronic and zero-point Energies= -3615.008845 $E_0 + E_{ZPE}$
Sum of electronic and thermal Energies= -3614.963717 $E_0 + E_{tot}$
Sum of electronic and thermal Enthalpies= -3614.962709 $E_0 + H_{corr}$
Sum of electronic and thermal Free Energies= -3615.091171 $E_0 + G_{corr}$
Zero-point correction (*unscaled*) = 0.618215
Number of imaginary vibrational frequencies = 0

H_2

Charge 0; multiplicity 1

H	0.52510600	0.13564200	4.20036800
H	0.18351900	-0.12813100	4.82122400

DFT TPSSh; def2-TZVPP basis set on Ni, def2-SVP on the other atoms; 2,2,2-trifluoroethanol, SMD model
Total electronic energy= -1.174981 E_0
Sum of electronic and zero-point Energies=-1.164884 $E_0 + E_{ZPE}$
Sum of electronic and thermal Energies= -1.162366 $E_0 + E_{tot}$
Sum of electronic and thermal Enthalpies= -1.161358 $E_0 + H_{corr}$
Sum of electronic and thermal Free Energies= -1.177407 $E_0 + G_{corr}$
Zero-point correction (*unscaled*) = 0.010097
Number of imaginary vibrational frequencies = 0

3-R

Charge 1; multiplicity 1

Ni	0.37037000	-0.18556600	0.84264500
C	3.10658500	0.89603800	0.83165300
P	-1.34328800	-1.51614000	0.59462400
P	-0.55988900	1.26467500	-0.41739800
C	-2.46903800	-0.76929000	-0.65522500
C	-2.05699700	0.49398800	-1.17788000
C	3.84629200	-0.33839000	0.27228400
H	3.96108700	-1.06734400	1.09132500
H	4.85898100	-0.04133600	-0.04522600
N	-3.56743000	-1.37091200	-1.06866800
N	-2.71333000	1.10163800	-2.14539400
C	-4.28280600	-0.75626600	-2.05059100
C	-5.47385100	-1.35618000	-2.53850900
H	-5.80053400	-2.30010700	-2.09772100
C	-3.83616300	0.49119900	-2.61451500
C	-4.58396600	1.09423800	-3.66023900
C	-6.18221400	-0.74270300	-3.55234400
C	-5.73520200	0.48374900	-4.11605200
H	-4.22772200	2.03781600	-4.07817300
H	-6.31523800	0.94515500	-4.91860500
H	-7.09777400	-1.20211700	-3.93198800
O	1.88164700	0.52563700	1.42445400
C	-0.82570300	-3.21366000	-0.04667400
C	-1.98928500	-4.21531100	0.00823600
C	-0.33122200	-3.03947300	-1.49260300
C	0.32718600	-3.67371700	0.86617700
H	-2.31390400	-4.41209000	1.04131400
H	-2.85512800	-3.86723900	-0.57438500
H	-1.64755400	-5.17216700	-0.42254500
H	0.43569800	-2.25334100	-1.57313000
H	0.12009600	-3.98537900	-1.83540300
H	-1.15654200	-2.79099600	-2.17783800
H	0.68990900	-4.65703400	0.52304900
H	1.17578200	-2.97058300	0.83810600
H	0.00045000	-3.78085100	1.91292400
C	-2.37511000	-1.76319600	2.08566800
H	-3.28592300	-2.32385800	1.82854100
H	-1.79437100	-2.31341800	2.84075800
H	-2.65122500	-0.77888400	2.48982000
C	-1.14581100	2.77921200	0.55055900
C	-1.88439700	3.75722600	-0.37943800
C	-2.07888600	2.28111500	1.66655300
C	0.09139700	3.46356900	1.15702200
H	-1.22936200	4.14241700	-1.17568000
H	-2.77260200	3.30400500	-0.84230300
H	-2.21635700	4.61750500	0.22625400
H	-1.55777300	1.58839200	2.34661000
H	-2.41864800	3.14688900	2.25887600
H	-2.97299600	1.77814800	1.26431700

H	-0.24704300	4.33525100	1.74157800
H	0.64300800	2.79220800	1.83119100
H	0.78146600	3.82927900	0.38090100
C	0.43530100	1.82442200	-1.84036500
H	-0.18254700	2.47007000	-2.48248000
H	1.31555500	2.38018200	-1.48802100
H	0.75769100	0.94317400	-2.41299500
C	3.10795200	-0.98480900	-0.89474800
H	3.21900100	-0.41538700	-1.82923800
H	2.01715800	-1.00780300	-0.67606100
C	3.48571800	-2.42149400	-1.13917100
O	3.92436600	-3.18793600	-0.29894300
O	3.23820100	-2.80528200	-2.40268300
H	3.45674500	-3.75671300	-2.48694100
H	0.52510600	0.13564200	4.20036800
H	0.18351900	-0.12813100	4.82122400
C	3.97346500	1.62499700	1.85084000
C	4.77054100	2.71281800	1.45924800
C	4.01784400	1.20133000	3.18986000
C	5.59721400	3.36325800	2.38313300
H	4.74072200	3.05626500	0.42063800
C	4.84107200	1.85215700	4.11559500
H	3.39881200	0.35914400	3.50935600
C	5.63446000	2.93471800	3.71528400
H	6.20866600	4.21124900	2.06264100
H	4.86354700	1.51245700	5.15477300
H	6.27627300	3.44378900	4.43930200
H	2.91676900	1.59488600	-0.00473600

DFT TPSSh; def2-TZVPP basis set on Ni, def2-SVP on the other atoms; 2,2,2-trifluoroethanol, SMD model
Total electronic energy= -3616.799367 E_0
Sum of electronic and zero-point Energies= -3616.168750 $E_0 + E_{ZPE}$
Sum of electronic and thermal Energies= -3616.119610 $E_0 + E_{tot}$
Sum of electronic and thermal Enthalpies= -3616.118602 $E_0 + H_{corr}$
Sum of electronic and thermal Free Energies= -3616.256492 $E_0 + G_{corr}$
Zero-point correction (*unscaled*) = 0.630616
Number of imaginary vibrational frequencies = 0

TS2-R

Charge 1; multiplicity 1

Ni	0.38976400	-0.23549500	0.98845200
C	3.11314700	0.87912600	0.79485100
P	-1.35116800	-1.54265400	0.66999900
P	-0.47656600	1.23662200	-0.30229300
C	-2.40928000	-0.76670600	-0.62026800
C	-1.94587400	0.48292700	-1.13096200
C	3.87983900	-0.36052400	0.28090700
H	4.04391800	-1.03624400	1.13634200
H	4.87095000	-0.05195600	-0.08922000
N	-3.50525500	-1.34319000	-1.07423600
N	-2.54229600	1.09615800	-2.13331900
C	-4.16183500	-0.71987300	-2.09108400
C	-5.34681200	-1.29275800	-2.62439900
H	-5.72017100	-2.22060900	-2.18661800
C	-3.65598300	0.50732500	-2.64920700
C	-4.33653900	1.11437500	-3.73761100
C	-5.99003400	-0.67519800	-3.67845900
C	-5.48181400	0.52875700	-4.23882200
H	-3.93492200	2.04138000	-4.15143800
H	-6.01012300	0.99327300	-5.07459700
H	-6.90011100	-1.11443800	-4.09351700
O	1.94436200	0.49065100	1.47626900
C	-0.82930700	-3.23559700	0.01293100
C	-2.00064000	-4.22937100	0.03650500
C	-0.31382300	-3.04894500	-1.42387800
C	0.30593600	-3.71707500	0.93634000
H	-2.34086400	-4.44149300	1.06132800
H	-2.85559300	-3.86567900	-0.55272800
H	-1.65967000	-5.18128000	-0.40580800
H	0.44986300	-2.25877700	-1.48861700
H	0.14705000	-3.99035400	-1.76617100
H	-1.13039000	-2.80004000	-2.11917000
H	0.66446000	-4.69934000	0.58564200
H	1.16153100	-3.02217100	0.93017000
H	-0.03712900	-3.83607400	1.97636100
C	-2.47748200	-1.81951300	2.08674100
H	-3.39589800	-2.31655100	1.74104500
H	-1.97574200	-2.44263300	2.84089800
H	-2.73326900	-0.84737300	2.53196800
C	-1.11062200	2.72988300	0.67321100
C	-1.79544500	3.73210500	-0.27156800
C	-2.10370000	2.20977100	1.72519500
C	0.09283000	3.39600100	1.36230100
H	-1.09609100	4.13840100	-1.01801000
H	-2.65482600	3.29129900	-0.79709200
H	-2.16402200	4.57604500	0.33595800
H	-1.62110900	1.49935600	2.41551400
H	-2.47256200	3.06245200	2.31909800
H	-2.97616700	1.71917900	1.26430100

H	-0.27519100	4.25845500	1.94278300
H	0.60263000	2.70965500	2.05418400
H	0.82871400	3.77277700	0.63497600
C	0.53507900	1.85038800	-1.69082700
H	-0.11438400	2.42182600	-2.37073200
H	1.34337600	2.49448100	-1.31850500
H	0.96049600	0.99199100	-2.22977300
C	3.12048100	-1.09248100	-0.82049600
H	3.20204000	-0.58635600	-1.79344900
H	2.03705200	-1.10793300	-0.56966400
C	3.50380000	-2.53967800	-0.97888900
O	3.93993300	-3.25454400	-0.09302200
O	3.26482800	-2.99766800	-2.21901900
H	3.48717000	-3.95136500	-2.24398100
H	0.63324600	-1.25250500	3.15476300
H	0.05696000	-0.76913900	3.26459800
C	3.99922500	1.71042700	1.71615800
C	4.79771900	2.74109500	1.19307600
C	4.06314000	1.43928400	3.09299400
C	5.64370500	3.48358600	2.02513800
H	4.75375200	2.96736500	0.12317600
C	4.90679100	2.18202600	3.92717600
H	3.44142600	0.64471700	3.51303500
C	5.70075600	3.20610000	3.39631700
H	6.25515700	4.28538900	1.60206600
H	4.94395400	1.96040100	4.99743700
H	6.35788200	3.78762100	4.04848300
H	2.86069800	1.50988900	-0.07731400

DFT TPSSh; def2-TZVPP basis set on Ni, def2-SVP on the other atoms; 2,2,2-trifluoroethanol, SMD model
Total electronic energy= -3616.797862 E_0
Sum of electronic and zero-point Energies= -3616.165095 $E_0 + E_{ZPE}$
Sum of electronic and thermal Energies= -3616.118347 $E_0 + E_{tot}$
Sum of electronic and thermal Enthalpies= -3616.117339 $E_0 + H_{corr}$
Sum of electronic and thermal Free Energies= -3616.247644 $E_0 + G_{corr}$
Zero-point correction (*unscaled*) = 0.632767
Number of imaginary vibrational frequencies = 1; 288i

4-R

Charge 1; multiplicity 1

Ni	0.41877900	-0.24094700	0.98335600
C	3.05875800	1.04400800	0.84283300
P	-1.33319800	-1.55163300	0.63478500
P	-0.42946200	1.23996500	-0.35822000
C	-2.39051800	-0.73708200	-0.63846700
C	-1.88888200	0.47944600	-1.19154200
C	3.71937400	-0.31294900	0.51362200
H	3.71899700	-0.93997700	1.42072800
H	4.77290600	-0.14162600	0.23808300
N	-3.51278200	-1.28443300	-1.06345300
N	-2.45882300	1.06485700	-2.22660900
C	-4.14943200	-0.67946100	-2.10330900
C	-5.36176200	-1.22335500	-2.60510400
H	-5.77763300	-2.10843300	-2.11960500
C	-3.58910800	0.49266200	-2.72296000
C	-4.24024600	1.06999200	-3.84535000
C	-5.97639500	-0.63369700	-3.69161000
C	-5.41193300	0.51234600	-4.31626100
H	-3.79680200	1.95479300	-4.30625100
H	-5.91870900	0.95517100	-5.17681500
H	-6.90685700	-1.05102800	-4.08343900
O	1.79003700	0.91920600	1.44129300
C	-0.94160100	-3.27564100	-0.04947200
C	-2.20705800	-4.15088500	-0.05026600
C	-0.40168000	-3.11237700	-1.48044600
C	0.12629100	-3.91130300	0.85942600
H	-2.55690800	-4.36638700	0.97072900
H	-3.02788500	-3.68669500	-0.61547500
H	-1.95922100	-5.11385100	-0.52853400
H	0.45795800	-2.42623100	-1.52152300
H	-0.06288400	-4.09538700	-1.84786400
H	-1.17633200	-2.74448900	-2.17106100
H	0.28884000	-4.95198200	0.53246500
H	1.09164400	-3.38760900	0.80031000
H	-0.19484400	-3.94120300	1.91335400
C	-2.41345800	-1.78014400	2.09434400
H	-3.37466800	-2.22034200	1.79157200
H	-1.90961700	-2.43964700	2.81656700
H	-2.58811000	-0.80091200	2.56220800
C	-1.11053600	2.72405800	0.60932400
C	-2.10897500	3.52246400	-0.24527500
C	-1.80655900	2.16224400	1.85997600
C	0.06742400	3.63046100	1.00687800
H	-1.66754000	3.86240900	-1.19480100
H	-3.02306500	2.95228200	-0.46441600
H	-2.40157600	4.41917000	0.32730100
H	-1.09468800	1.62799500	2.51011100
H	-2.24082600	2.99541100	2.43745800
H	-2.62468200	1.47350100	1.59373500

H	-0.32263900	4.44009300	1.64654700
H	0.83444500	3.07823700	1.56697000
H	0.53709500	4.09763400	0.12724900
C	0.58060600	1.87118600	-1.73843700
H	-0.03899600	2.53940500	-2.35511400
H	1.45007500	2.41873700	-1.35131100
H	0.91902500	1.02139700	-2.34843600
C	3.01501500	-1.03843800	-0.62765700
H	3.14284400	-0.52038400	-1.58926000
H	1.91606100	-1.05916900	-0.43618200
C	3.42005300	-2.47915800	-0.78173900
O	3.82237500	-3.19645900	0.11855000
O	3.24984300	-2.92715200	-2.03632300
H	3.48160000	-3.87871700	-2.05854700
H	1.34829500	-1.08119300	2.20023000
H	0.69537100	-1.51998000	2.17912400
C	3.96096700	1.84409400	1.78020000
C	4.88200700	2.76915700	1.26154500
C	3.91507500	1.64612100	3.17055900
C	5.74357800	3.47534400	2.10940100
H	4.92284000	2.94045400	0.18139400
C	4.77351700	2.35288000	4.02051200
H	3.19583300	0.93765600	3.58874400
C	5.69198700	3.26926900	3.49317900
H	6.45230000	4.19367600	1.68801500
H	4.72470600	2.18786400	5.10053300
H	6.36075900	3.82309800	4.15753800
H	2.99682200	1.61392800	-0.10702200

DFT TPSSh; def2-TZVPP basis set on Ni, def2-SVP on the other atoms; 2,2,2-trifluoroethanol, SMD model
Total electronic energy= -3616.800638 E_0
Sum of electronic and zero-point Energies= -3616.164762 $E_0 + E_{ZPE}$
Sum of electronic and thermal Energies= -3616.118509 $E_0 + E_{tot}$
Sum of electronic and thermal Enthalpies= -3616.117502 $E_0 + H_{corr}$
Sum of electronic and thermal Free Energies= -3616.246409 $E_0 + G_{corr}$
Zero-point correction (*unscaled*) = 0.635876
Number of imaginary vibrational frequencies = 0

TS3-R

Charge 1; multiplicity 1

Ni	0.30672100	-0.21535600	0.97016400
C	3.04703200	0.99024500	1.11284000
P	-1.36079200	-1.55946300	0.63287100
P	-0.52748000	1.21100600	-0.50225700
C	-2.48843400	-0.78670100	-0.60932500
C	-2.04853800	0.43604000	-1.20428000
C	3.72511900	-0.29970600	0.61500000
H	3.76291600	-1.02302300	1.44615700
H	4.76772100	-0.06519100	0.34725400
N	-3.62205700	-1.36397500	-0.95706800
N	-2.70659900	1.00246500	-2.19781100
C	-4.34320700	-0.78043700	-1.95297900
C	-5.57278600	-1.35687400	-2.36898600
H	-5.92953600	-2.25237600	-1.85620900
C	-3.85825000	0.40511400	-2.60864800
C	-4.60288600	0.96567500	-3.68048800
C	-6.27814700	-0.78385100	-3.40824900
C	-5.78992100	0.37723700	-4.06843200
H	-4.21633000	1.86141100	-4.17070200
H	-6.36769700	0.80658800	-4.89022700
H	-7.22255500	-1.22571400	-3.73438900
O	1.79223800	0.74833100	1.74839900
C	-0.89887700	-3.25868500	-0.05748000
C	-2.12398800	-4.18970300	-0.05830400
C	-0.37492500	-3.05831100	-1.48984300
C	0.20143700	-3.84843500	0.84261000
H	-2.46427100	-4.41956600	0.96286700
H	-2.96509500	-3.76329400	-0.62364400
H	-1.83388300	-5.14087300	-0.53619100
H	0.45010900	-2.33041800	-1.52784400
H	0.00870300	-4.02020300	-1.86875700
H	-1.17045400	-2.72111700	-2.17266300
H	0.42232800	-4.87297900	0.49904400
H	1.13379900	-3.26711300	0.79392000
H	-0.11793500	-3.91042500	1.89529200
C	-2.39929800	-1.81621400	2.11573300
H	-3.34148500	-2.31015400	1.83683700
H	-1.84870300	-2.43276200	2.84123600
H	-2.61677400	-0.83662900	2.56554800
C	-1.08485100	2.81072400	0.34154000
C	-1.90697100	3.70156000	-0.60112700
C	-1.92277800	2.39622400	1.56335200
C	0.18355200	3.55680900	0.79513900
H	-1.33832600	3.99043900	-1.49849600
H	-2.84275000	3.22068700	-0.91954400
H	-2.16781000	4.62684700	-0.05904500
H	-1.33823300	1.76859800	2.25631000
H	-2.24065900	3.30092800	2.10823500
H	-2.83059200	1.84437400	1.27010600

H	-0.12297600	4.45990800	1.34955500
H	0.80472900	2.94011800	1.46115900
H	0.79464300	3.88370600	-0.06061700
C	0.45412400	1.65365500	-1.98030500
H	-0.13596600	2.30926000	-2.63795600
H	1.37922600	2.16347100	-1.67693300
H	0.70696700	0.72997800	-2.52070700
C	3.02635700	-0.91885800	-0.59195500
H	3.16192700	-0.31791100	-1.50284000
H	1.92651600	-0.96460700	-0.41729600
C	3.44268800	-2.33916600	-0.86817600
O	3.89254100	-3.11349900	-0.04142000
O	3.22339500	-2.69463200	-2.14445500
H	3.46351700	-3.63868300	-2.25116200
H	1.45507700	-0.51878900	2.10689600
H	0.81598600	-1.25642900	2.07558000
C	3.94862400	1.72640600	2.09508000
C	4.81540500	2.73129400	1.63507000
C	3.95907500	1.39547600	3.46017400
C	5.68117000	3.38730300	2.51777400
H	4.81021500	3.00427600	0.57546400
C	4.82186400	2.05307700	4.34445000
H	3.28150000	0.62430400	3.83457600
C	5.68695700	3.04974800	3.87643500
H	6.34785600	4.16918100	2.14393800
H	4.81746000	1.78649700	5.40493300
H	6.35899700	3.56429400	4.56837200
H	2.87928000	1.65099600	0.24204400

DFT TPSSh; def2-TZVPP basis set on Ni, def2-SVP on the other atoms;<br>2,2,2-trifluoroethanol, SMD model
Total electronic energy= -3616.788537 E_0
Sum of electronic and zero-point Energies= -3616.154292 $E_0 + E_{ZPE}$
Sum of electronic and thermal Energies= -3616.108749 $E_0 + E_{tot}$
Sum of electronic and thermal Enthalpies= -3616.107741 $E_0 + H_{corr}$
Sum of electronic and thermal Free Energies= -3616.236322 $E_0 + G_{corr}$
Zero-point correction (*unscaled*) = 0.634245
Number of imaginary vibrational frequencies = 1; 1189i

5-R

Charge 1; multiplicity 1

Ni	0.30411900	-0.32197700	1.03194800
C	3.14155900	0.91129300	1.08555300
P	-1.35650100	-1.57389000	0.67895000
P	-0.39128700	1.17488700	-0.49182100
C	-2.43609500	-0.75877100	-0.59512000
C	-1.92620300	0.42906600	-1.21305200
C	3.85862800	-0.32596900	0.53842900
H	4.02270200	-1.04170200	1.36037400
H	4.85285900	-0.01699300	0.17868400
N	-3.59854100	-1.27800200	-0.93937600
N	-2.54424600	0.99666400	-2.23232400
C	-4.27924800	-0.68408100	-1.95868000
C	-5.53713700	-1.20029000	-2.36917200
H	-5.95034400	-2.05696700	-1.83277100
C	-3.72118500	0.44916600	-2.64366600
C	-4.42142100	1.01743900	-3.74112400
C	-6.19937800	-0.62036400	-3.43316900
C	-5.63778300	0.48790300	-4.12414500
H	-3.97880700	1.87367500	-4.25425700
H	-6.18168000	0.92449100	-4.96511300
H	-7.16560200	-1.01619900	-3.75470000
O	1.91272300	0.52146700	1.78421800
C	-0.95139100	-3.27806300	-0.04965900
C	-2.21346400	-4.15671500	-0.10327800
C	-0.39019500	-3.06304600	-1.46612500
C	0.10559300	-3.95202100	0.84269000
H	-2.57900200	-4.40948500	0.90382400
H	-3.02837900	-3.67485000	-0.66263800
H	-1.95870600	-5.10189700	-0.61247900
H	0.47112100	-2.37711300	-1.46517100
H	-0.04705900	-4.03099900	-1.86848600
H	-1.15276500	-2.66548600	-2.15437800
H	0.27357300	-4.97744100	0.47146500
H	1.06880300	-3.42042300	0.81997200
H	-0.22671200	-4.02647700	1.89052100
C	-2.48940100	-1.84399100	2.09103100
H	-3.44067100	-2.27070000	1.74197300
H	-2.01357400	-2.51972700	2.81650400
H	-2.67965400	-0.87494800	2.57559300
C	-0.99059300	2.79142000	0.30590300
C	-1.65514800	3.73729400	-0.70358400
C	-1.98395100	2.39668800	1.41230000
C	0.24099700	3.47548700	0.92624800
H	-0.95163500	4.06295600	-1.48561100
H	-2.52874300	3.28027000	-1.19030800
H	-1.99659100	4.63999600	-0.16767700
H	-1.51945900	1.71426700	2.14375300
H	-2.30933800	3.30241400	1.95168200
H	-2.88335400	1.90844200	1.00302700

H	-0.08229400	4.40365400	1.42785100
H	0.72987000	2.83740600	1.67735500
H	0.98175600	3.75355300	0.16013200
C	0.56611300	1.64061100	-1.98696100
H	-0.05026400	2.24373000	-2.66954500
H	1.46297200	2.20626800	-1.69587400
H	0.87541000	0.71870000	-2.50144400
C	3.09117100	-1.00692800	-0.59209100
H	3.15529900	-0.44594800	-1.53512100
H	2.00803800	-1.06235900	-0.33662200
C	3.50783400	-2.43417400	-0.82852200
O	3.98112000	-3.17579900	0.01505600
O	3.25393400	-2.83719200	-2.08334000
H	3.49208200	-3.78454600	-2.16071700
H	2.15470500	0.02385500	2.58586800
H	0.41211800	-1.41659700	2.03772700
C	3.99572300	1.74924500	2.01663100
C	4.18581300	3.11594100	1.75556300
C	4.60949900	1.18231000	3.14852600
C	4.97335900	3.90169300	2.60495000
H	3.71648200	3.56721900	0.87718800
C	5.38854700	1.96882000	4.00268400
H	4.47882700	0.11862200	3.36896300
C	5.57440400	3.33024000	3.73178000
H	5.11507400	4.96322600	2.38604600
H	5.85686600	1.51550000	4.88017500
H	6.18758600	3.94318600	4.39749000
H	2.78520500	1.54001600	0.25904300

DFT TPSSh; def2-TZVPP basis set on Ni, def2-SVP on the other atoms; 2,2,2-trifluoroethanol, SMD model
Total electronic energy= -3616.822832 E_0
Sum of electronic and zero-point Energies= -3616.184309 $E_0 + E_{ZPE}$
Sum of electronic and thermal Energies= -3616.138004 $E_0 + E_{tot}$
Sum of electronic and thermal Enthalpies= -3616.136996 $E_0 + H_{corr}$
Sum of electronic and thermal Free Energies= -3616.267775 $E_0 + G_{corr}$
Zero-point correction (*unscaled*) = 0.638523
Number of imaginary vibrational frequencies = 0

6-R

Charge 0; multiplicity 1

C	3.02882500	0.69455000	1.39859600
C	3.83778800	-0.20969700	0.45387800
H	4.49443400	-0.85561500	1.06275800
H	4.49778400	0.40921900	-0.17360700
O	2.16999400	-0.08010800	2.22883700
C	2.94107300	-1.07005600	-0.43077500
H	2.32408800	-0.45188800	-1.10522100
H	2.22715100	-1.64872000	0.17953000
C	3.70607700	-2.05065800	-1.28033600
O	4.91145500	-2.22545100	-1.25010000
O	2.89809700	-2.74387200	-2.10063700
H	3.44661900	-3.36906800	-2.61694200
H	2.71373700	-0.74772300	2.68303000
C	3.92548200	1.63428000	2.20204900
C	4.73025400	2.58249900	1.54361200
C	3.95605900	1.58715600	3.60381900
C	5.55328400	3.44937900	2.26882100
H	4.70881500	2.65007400	0.45189200
C	4.78355900	2.45223200	4.33201500
H	3.31745900	0.87434100	4.13024700
C	5.58670800	3.38473000	3.66814200
H	6.16949000	4.18120300	1.73925600
H	4.79467400	2.39770700	5.42416200
H	6.23076400	4.06177900	4.23557300
H	2.34637100	1.31472900	0.79220900

DFT TPSSh; def2-TZVPP basis set on Ni, def2-SVP on the other atoms; 2,2,2-trifluoroethanol, SMD model
Total electronic energy= -613.583577 E_0
Sum of electronic and zero-point Energies= -613.379452 $E_0 + E_{ZPE}$
Sum of electronic and thermal Energies= -613.365598 $E_0 + E_{tot}$
Sum of electronic and thermal Enthalpies= -613.364591 $E_0 + H_{corr}$
Sum of electronic and thermal Free Energies= -613.423720 $E_0 + G_{corr}$
Zero-point correction (*unscaled*) = 0.204125
Number of imaginary vibrational frequencies = 0

1-S

Charge 1; multiplicity 1

Ni	0.49302000	-0.08073500	1.07476300
C	3.28515200	0.65052200	0.77886600
P	-1.10820100	-1.42463100	1.39747800
P	-0.68148000	1.06713000	-0.45283600
C	4.34067500	1.65651800	0.99955000
C	-2.55219700	-0.86182100	0.37222200
C	-2.31092100	0.19314700	-0.56687600
C	5.66716000	1.43634300	0.57121800
C	4.01814300	2.87112300	1.64467200
C	6.64369300	2.41212700	0.77837300
H	5.93989000	0.50648300	0.06999400
C	4.99831500	3.83796200	1.85501200
H	2.99381500	3.04117800	1.97999500
C	6.31254800	3.61105300	1.42068300
H	7.66712000	2.23553900	0.43981900
H	4.74228100	4.77267400	2.35935400
H	7.08020400	4.37137400	1.58515400
H	0.97013900	-0.98731900	2.15839600
C	3.57832600	-0.69722100	0.17717600
H	2.82280600	-1.38654100	0.58651100
H	4.57373400	-1.05642500	0.47269000
N	-3.73306500	-1.43874000	0.48199500
N	-3.22797000	0.56225300	-1.44187100
C	-4.71282000	-1.04060000	-0.37603500
C	-6.00554400	-1.62344100	-0.29644700
H	-6.19537400	-2.36733200	0.48002200
C	-4.44216600	-0.05172600	-1.38296000
C	-5.46294200	0.30529100	-2.30394300
C	-6.98104900	-1.24823300	-1.19882400
C	-6.70740400	-0.28542300	-2.20837000
H	-5.23844100	1.05271200	-3.06765100
H	-7.49672300	-0.01042600	-2.91189600
H	-7.97609700	-1.69595800	-1.14343400
O	2.10906700	0.93625400	1.09481100
C	-0.77393700	-3.19715900	0.81146400
C	-1.95985900	-4.11473400	1.15409200
C	-0.55259800	-3.14525300	-0.71045200
C	0.49477700	-3.71654400	1.50990300
H	-2.08029400	-4.23656800	2.24167300
H	-2.90717600	-3.74361700	0.73721700
H	-1.76334200	-5.11262400	0.72611300
H	0.25119200	-2.44062500	-0.97789600
H	-0.25397400	-4.14534000	-1.06694800
H	-1.46811200	-2.85507200	-1.25049500
H	0.62612400	-4.78029000	1.24804200
H	1.39407500	-3.17495400	1.18289100
H	0.42148800	-3.64904500	2.60709800
C	-1.79150200	-1.52676200	3.09140600
H	-2.76586200	-2.03591000	3.08137000

H	-1.08518000	-2.06979400	3.73636300
H	-1.91635100	-0.50482400	3.47891200
C	-1.13448300	2.83693600	0.06434100
C	-1.92619000	3.56977900	-1.02862900
C	-1.95988700	2.74150000	1.35913600
C	0.18563500	3.57769700	0.34245400
H	-1.32122200	3.72830600	-1.93481900
H	-2.84081200	3.02756500	-1.30984000
H	-2.21900300	4.56311700	-0.64644300
H	-1.42464000	2.17495500	2.13940000
H	-2.14454900	3.75771300	1.74686600
H	-2.93849900	2.26403800	1.19098500
H	-0.04033600	4.63040800	0.58377700
H	0.72205900	3.13965800	1.19664400
H	0.85671200	3.57238500	-0.53169500
C	-0.12750100	1.15902600	-2.19989400
H	-0.93122600	1.54699800	-2.84266600
H	0.75480000	1.81198300	-2.27033400
H	0.14559500	0.14710300	-2.53359600
C	3.46368300	-0.68225000	-1.35449000
H	4.27737400	-0.11235100	-1.82532000
H	2.51831300	-0.20499100	-1.66642100
C	3.43934100	-2.07923100	-1.92819800
O	3.09862200	-3.07974700	-1.32297700
O	3.80809200	-2.09632800	-3.21499000
H	3.73098800	-3.01354700	-3.55011800

DFT TPSSh; def2-TZVPP basis set on Ni, def2-SVP on the other atoms; 2,2,2-trifluoroethanol, SMD model
Total electronic energy= -3615.625862 E_0
Sum of electronic and zero-point Energies= -3615.010783 $E_0 + E_{ZPE}$
Sum of electronic and thermal Energies= -3614.964915 $E_0 + E_{tot}$
Sum of electronic and thermal Enthalpies= -3614.963908 $E_0 + H_{corr}$
Sum of electronic and thermal Free Energies= -3615.094300 $E_0 + G_{corr}$
Zero-point correction (*unscaled*) = 0.615079
Number of imaginary vibrational frequencies = 0

TS1-S

Charge 1; multiplicity 1

Ni	0.55894500	0.14757300	0.21170700
C	3.09800100	0.56441300	0.02412600
P	-0.82997700	-1.13427100	1.14699500
P	-1.01776000	1.11445800	-1.03785000
C	3.87146800	1.17044600	1.12627900
C	-2.51741200	-0.70410900	0.49352900
C	-2.58336100	0.23305700	-0.58910700
C	4.99570800	0.52768600	1.68781300
C	3.48370900	2.43637200	1.61809900
C	5.71242900	1.14007700	2.71692400
H	5.31049200	-0.45347700	1.32941400
C	4.20372900	3.04354000	2.64378900
H	2.61628800	2.93363100	1.18100700
C	5.31866100	2.39559600	3.19566900
H	6.57970900	0.63577600	3.14893000
H	3.89991900	4.02408700	3.01748300
H	5.88154700	2.87198700	4.00229900
H	1.36397400	-0.64913600	1.19417200
C	3.58484200	-0.67583500	-0.68710800
H	2.71601500	-1.14132800	-1.17226900
H	4.01528200	-1.40068500	0.01653200
N	-3.59894400	-1.27475400	0.98661400
N	-3.71912400	0.48723500	-1.21222700
C	-4.79074500	-0.99282600	0.39104000
C	-5.98733500	-1.57757900	0.88426300
H	-5.92879700	-2.22748800	1.75979600
C	-4.84485200	-0.12692400	-0.75426300
C	-6.09073900	0.10835400	-1.39452600
C	-7.18532900	-1.32269400	0.24653000
C	-7.23641800	-0.48160700	-0.89850100
H	-6.11257600	0.76332800	-2.26798200
H	-8.19737500	-0.30067200	-1.38590300
H	-8.10786500	-1.77342000	0.61988200
O	2.09218300	1.17278100	-0.44205300
C	-0.60307900	-2.97256800	0.74329600
C	-1.63071900	-3.83012500	1.50207200
C	-0.77805300	-3.13709600	-0.77620300
C	0.81750500	-3.39094800	1.15892100
H	-1.47267000	-3.78768200	2.59082600
H	-2.66534000	-3.52729000	1.28715000
H	-1.50695200	-4.88047700	1.18770400
H	-0.07460300	-2.49859800	-1.33571600
H	-0.57566600	-4.18574500	-1.05162700
H	-1.80258200	-2.89664100	-1.10255300
H	0.92478100	-4.47654400	0.99422400
H	1.58760600	-2.87834500	0.56374100
H	1.01018600	-3.19349400	2.22560800
C	-1.01306700	-0.98985000	2.96121500
H	-1.93279700	-1.49431500	3.29103000

H	-0.13691600	-1.43719100	3.45296000
H	-1.06638700	0.07704000	3.22352500
C	-1.35919200	2.94221900	-0.66502200
C	-2.50014700	3.50905000	-1.52235900
C	-1.70487500	3.04231500	0.83071400
C	-0.05847600	3.71280300	-0.95658100
H	-2.25017000	3.49851600	-2.59465200
H	-3.44021600	2.95723400	-1.37896600
H	-2.67045800	4.56017000	-1.23129200
H	-0.90389600	2.61957100	1.45981200
H	-1.82730900	4.10360900	1.10596600
H	-2.64650000	2.52278200	1.07160400
H	-0.22815200	4.78432100	-0.75531700
H	0.77085600	3.37251000	-0.31938900
H	0.24983900	3.61604900	-2.00982300
C	-0.93255500	0.95022300	-2.86248500
H	-1.87651000	1.26303200	-3.33232600
H	-0.10223700	1.56453600	-3.24170000
H	-0.73395600	-0.10403200	-3.10706100
C	4.62381000	-0.31155200	-1.75843200
H	5.52318100	0.15150800	-1.32078900
H	4.21533000	0.41950700	-2.47554000
C	5.06103400	-1.52820800	-2.53820900
O	4.64931400	-2.65973200	-2.36141800
O	5.97198900	-1.21952200	-3.47113900
H	6.21786100	-2.03858000	-3.94861700

DFT TPSSh; def2-TZVPP basis set on Ni, def2-SVP on the other atoms; 2,2,2-trifluoroethanol, SMD model
Total electronic energy= -3615.611400 E_0
Sum of electronic and zero-point Energies= -3614.997182 $E_0 + E_{ZPE}$
Sum of electronic and thermal Energies= -3614.951840 $E_0 + E_{tot}$
Sum of electronic and thermal Enthalpies= -3614.950832 $E_0 + H_{corr}$
Sum of electronic and thermal Free Energies= -3615.082527 $E_0 + G_{corr}$
Zero-point correction (*unscaled*) = 0.614218
Number of imaginary vibrational frequencies = 1; 62i

2-S

Charge 1; multiplicity 1

Ni	0.60395100	0.12412400	-0.02610600
C	2.75870000	0.15539400	-0.03855300
P	-0.79591600	-1.13091800	1.08468300
P	-1.01950100	1.15208600	-1.01015100
C	3.55322000	0.91989400	1.01692200
C	-2.48883200	-0.71247900	0.46923000
C	-2.57222000	0.25300700	-0.58272800
C	4.02029500	0.27495400	2.17415700
C	3.85979900	2.27429000	0.81554700
C	4.77727300	0.97585800	3.11812100
H	3.78300400	-0.77941000	2.34367500
C	4.62255300	2.97345200	1.75862500
H	3.49982800	2.77606200	-0.08510800
C	5.08155900	2.32761400	2.91201700
H	5.12874300	0.46579700	4.01881900
H	4.85869500	4.02746400	1.58982100
H	5.67392700	2.87517000	3.64967200
H	2.01515300	-0.53327100	0.62335100
C	3.57733000	-0.90514600	-0.80471800
H	2.87997400	-1.51450600	-1.39822800
H	4.08059900	-1.57564100	-0.09166100
N	-3.56326000	-1.30539400	0.95222300
N	-3.71027500	0.53401500	-1.18624100
C	-4.75900200	-1.01044200	0.37208700
C	-5.94821100	-1.62084200	0.85175000
H	-5.87858000	-2.30310200	1.70135800
C	-4.82884500	-0.10148200	-0.74104100
C	-6.08123200	0.15105300	-1.36092900
C	-7.15251300	-1.34902100	0.23373700
C	-7.21852300	-0.46478600	-0.87776000
H	-6.11436700	0.83837900	-2.20865700
H	-8.18479500	-0.27155600	-1.34953400
H	-8.06922700	-1.81936500	0.59693800
O	2.00322000	0.95241000	-0.86627700
C	-0.56143700	-2.97753900	0.75462300
C	-1.53415800	-3.82402300	1.59106600
C	-0.79194600	-3.20663000	-0.74899800
C	0.88984400	-3.32598100	1.12906900
H	-1.33940000	-3.72286900	2.66991300
H	-2.58331000	-3.55854600	1.39778600
H	-1.39109700	-4.88503300	1.32380800
H	-0.13662200	-2.56576200	-1.36215400
H	-0.56289000	-4.25742200	-0.99311900
H	-1.83729800	-3.01455500	-1.03841800
H	1.04330600	-4.40821700	0.98183600
H	1.61498200	-2.79367100	0.49490900
H	1.10973400	-3.09750500	2.18415700
C	-0.88907900	-0.89058600	2.89570700
H	-1.75023600	-1.43461000	3.31062100

H	0.04245100	-1.24918500	3.35814000
H	-1.00082300	0.18431500	3.09965800
C	-1.27847000	2.93025000	-0.42315400
C	-2.40281500	3.59933900	-1.23119800
C	-1.63805800	2.87838700	1.07124100
C	0.04272200	3.69305400	-0.62238400
H	-2.13384200	3.70795800	-2.29303200
H	-3.35000300	3.04441300	-1.16401800
H	-2.56806700	4.61060700	-0.82213700
H	-0.85314400	2.37408600	1.65806200
H	-1.73364800	3.90925000	1.45106600
H	-2.59648300	2.36503600	1.24922500
H	-0.11789400	4.74787700	-0.34299900
H	0.84431300	3.28922900	0.01285600
H	0.38101300	3.67114100	-1.67033000
C	-0.93040300	1.14879600	-2.83300300
H	-1.87276900	1.51993500	-3.26145600
H	-0.09204100	1.78442400	-3.15340600
H	-0.75198900	0.11717100	-3.16974100
C	4.60007300	-0.25268900	-1.73074000
H	5.34384100	0.34139000	-1.17448500
H	4.10713000	0.44880600	-2.42536700
C	5.34656800	-1.25995600	-2.56692000
O	5.12624700	-2.45771700	-2.59262400
O	6.30362700	-0.68407600	-3.31124200
H	6.74261800	-1.37963700	-3.84250300

DFT TPSSh; def2-TZVPP basis set on Ni, def2-SVP on the other atoms; 2,2,2-trifluoroethanol, SMD model
Total electronic energy= -3615.628286 E_0
Sum of electronic and zero-point Energies= -3615.010242 $E_0 + E_{ZPE}$
Sum of electronic and thermal Energies= -3614.965036 $E_0 + E_{tot}$
Sum of electronic and thermal Enthalpies= -3614.964029 $E_0 + H_{corr}$
Sum of electronic and thermal Free Energies= -3615.092911 $E_0 + G_{corr}$
Zero-point correction (*unscaled*) = 0.618043
Number of imaginary vibrational frequencies = 0

3-S

Charge 1; multiplicity 1

Ni	0.42563700	-0.40492700	0.53980700
C	3.16278200	0.69080500	0.79597500
P	-1.33729300	-1.64639600	0.85637600
P	-0.82825500	1.19065700	-0.11653600
C	3.69042200	1.75293400	-0.16543200
C	-2.77111500	-0.80844700	0.06113400
C	-2.50342300	0.49323400	-0.46126600
C	4.35174200	2.88570900	0.34034000
C	3.55463000	1.61780000	-1.55629200
C	4.87131600	3.85685900	-0.52244700
H	4.45674000	3.01097500	1.42259300
C	4.07358500	2.58931300	-2.42210900
H	3.03148400	0.74773700	-1.95955200
C	4.73449600	3.71119300	-1.90895900
H	5.37949600	4.73330200	-0.11090800
H	3.95915000	2.46867500	-3.50301100
H	5.13728900	4.47045800	-2.58461800
H	2.80740800	1.20904300	1.70461300
C	4.28318400	-0.26852400	1.26779700
H	3.85886300	-0.92171400	2.04734000
H	5.07642100	0.32959700	1.74538200
N	-3.95946000	-1.37056200	-0.03962200
N	-3.40689000	1.18401500	-1.12592700
C	-4.92195900	-0.67441000	-0.70562100
C	-6.22090200	-1.22925700	-0.85207200
H	-6.42286900	-2.20647900	-0.40889700
C	-4.63506400	0.61570400	-1.27746700
C	-5.64946300	1.30582000	-1.99201200
C	-7.18795100	-0.53154100	-1.54785600
C	-6.90094000	0.73742200	-2.12137700
H	-5.41229100	2.28090900	-2.42212100
H	-7.68539000	1.26568000	-2.66822200
H	-8.18799000	-0.95567500	-1.66409700
O	2.13361500	-0.07508300	0.22006600
C	-1.02337400	-3.26466500	-0.05885300
C	-2.18987600	-4.24896400	0.10799300
C	-0.80680700	-2.91154600	-1.54034000
C	0.26949100	-3.84882700	0.54251100
H	-2.33278500	-4.53964200	1.16013000
H	-3.13417700	-3.83337800	-0.27298400
H	-1.96037000	-5.16428900	-0.46432400
H	0.01321900	-2.18407300	-1.66509500
H	-0.53701500	-3.82653400	-2.09352300
H	-1.71667100	-2.49422200	-2.00029300
H	0.53299600	-4.77081300	-0.00222800
H	1.11529200	-3.14595700	0.45051700
H	0.14681300	-4.10887200	1.60561400
C	-1.88923700	-2.03970800	2.55345300
H	-2.83947000	-2.59333900	2.51545600

H	-1.12179600	-2.64510400	3.05695300
H	-2.03379700	-1.10037000	3.10597400
C	-1.07142600	2.56457400	1.15538400
C	-1.91365400	3.69873800	0.54785300
C	-1.77437500	1.95600200	2.37919200
C	0.32202800	3.08209400	1.54902300
H	-1.39844800	4.18648100	-0.29354800
H	-2.89747300	3.34748800	0.20321500
H	-2.07713500	4.46101700	1.32859900
H	-1.19109100	1.12486100	2.80645900
H	-1.87351300	2.73372700	3.15455700
H	-2.78599300	1.59232700	2.13801600
H	0.19908000	3.91131600	2.26565500
H	0.91856600	2.29708800	2.03774600
H	0.88264300	3.46691500	0.68319400
C	-0.29929800	1.93932400	-1.69406300
H	-1.11273900	2.56328100	-2.09342900
H	0.60075700	2.54737900	-1.52881200
H	-0.07167700	1.13153700	-2.40432000
C	4.87609500	-1.12654500	0.15559700
H	5.35441300	-0.51790800	-0.62949200
H	4.09106900	-1.71170100	-0.35395700
C	5.90020900	-2.11125600	0.65662500
O	6.16599100	-2.32506700	1.82645200
O	6.50620500	-2.76282000	-0.35053000
H	7.14631600	-3.39687000	0.03232700
H	1.26012200	-0.76555300	3.93307700
H	1.07314600	-0.70185400	3.20172400

DFT TPSSh; def2-TZVPP basis set on Ni, def2-SVP on the other atoms; 2,2,2-trifluoroethanol, SMD model
Total electronic energy= -3616.795895 E_0
Sum of electronic and zero-point Energies= -3616.164306 $E_0 + E_{ZPE}$
Sum of electronic and thermal Energies= -3616.115805 $E_0 + E_{tot}$
Sum of electronic and thermal Enthalpies= -3616.114798 $E_0 + H_{corr}$
Sum of electronic and thermal Free Energies= -3616.250960 $E_0 + G_{corr}$
Zero-point correction (*unscaled*) = 0.631589
Number of imaginary vibrational frequencies = 0

TS2-S

Charge 1; multiplicity 1

Ni	0.42280500	-0.43947100	0.51832200
C	3.10892600	0.64102300	0.67154000
P	-1.35143000	-1.66378700	0.88784100
P	-0.81487100	1.12800900	-0.22787600
C	3.69643300	1.79540800	-0.13720200
C	-2.77596800	-0.84510300	0.05661600
C	-2.49214700	0.42113000	-0.53986500
C	4.35704900	2.84631100	0.52315700
C	3.62088700	1.82502300	-1.53907900
C	4.93264400	3.89795400	-0.19818700
H	4.41679300	2.84331000	1.61611300
C	4.19555900	2.87742000	-2.26367000
H	3.10132100	1.01961600	-2.06297400
C	4.85404300	3.91684000	-1.59661800
H	5.43875700	4.70862700	0.33326800
H	4.12733600	2.88474900	-3.35515400
H	5.30049800	4.73920300	-2.16197400
H	2.74336500	1.05961800	1.62605000
C	4.19019700	-0.39892100	1.05634500
H	3.73113100	-1.12000000	1.75240100
H	4.99296000	0.11477900	1.61054200
N	-3.97261200	-1.39502400	-0.00900600
N	-3.38722900	1.08320200	-1.24385400
C	-4.92798500	-0.72423400	-0.71056100
C	-6.23611300	-1.26620900	-0.81865500
H	-6.45128400	-2.21169600	-0.31671000
C	-4.62384000	0.52481500	-1.35933200
C	-5.63014800	1.18610400	-2.11156100
C	-7.19520600	-0.59650500	-1.55203800
C	-6.89080700	0.63064200	-2.20235500
H	-5.38003000	2.12984100	-2.60033300
H	-7.66920900	1.13731900	-2.77754200
H	-8.20231300	-1.01081000	-1.63894500
O	2.07191200	-0.01563100	-0.02111600
C	-1.05125500	-3.31119500	0.01907900
C	-2.21738700	-4.28740000	0.22815300
C	-0.84981800	-3.00523200	-1.47519700
C	0.24781100	-3.87728800	0.62439400
H	-2.34640200	-4.54998600	1.28941900
H	-3.16601400	-3.87899200	-0.15033400
H	-1.99911500	-5.21818600	-0.32328400
H	-0.03611100	-2.27673300	-1.63066000
H	-0.57843300	-3.93594600	-2.00068100
H	-1.76648300	-2.60931800	-1.94048200
H	0.49809000	-4.82352500	0.11617000
H	1.09556600	-3.18394600	0.48732700
H	0.14140200	-4.09002900	1.69991200
C	-1.91301900	-2.00162600	2.59341300
H	-2.86403900	-2.55442400	2.57260100

H	-1.14782100	-2.59078900	3.11931800
H	-2.05706800	-1.04415700	3.11446000
C	-1.07346800	2.56423200	0.97304300
C	-1.86063400	3.68900200	0.27998600
C	-1.84434400	2.03200900	2.19139000
C	0.31584100	3.06491800	1.40141700
H	-1.29519400	4.13262500	-0.55332100
H	-2.83350200	3.34197000	-0.09865100
H	-2.04661700	4.48525000	1.02080200
H	-1.30707200	1.20312100	2.67934600
H	-1.94894000	2.84584700	2.92803600
H	-2.85672100	1.68904400	1.92463100
H	0.18753600	3.93822800	2.06263100
H	0.86558700	2.29292100	1.96108900
H	0.92534400	3.38099800	0.54081200
C	-0.27964400	1.80531100	-1.83472700
H	-1.10301800	2.38397200	-2.27888300
H	0.60032800	2.44632700	-1.68671300
H	-0.01679800	0.96851800	-2.49756500
C	4.78019400	-1.15202500	-0.13117700
H	5.29433200	-0.47914100	-0.83742100
H	3.98874900	-1.65277500	-0.71520600
C	5.76348100	-2.21652800	0.28059800
O	6.00723900	-2.55125700	1.42675500
O	6.36155600	-2.78862600	-0.77822700
H	6.97844500	-3.47448100	-0.45016300
H	1.43184500	-0.80120300	3.00013800
H	0.77845800	-0.44561700	2.84502600

DFT TPSSh; def2-TZVPP basis set on Ni, def2-SVP on the other atoms; 2,2,2-trifluoroethanol, SMD model
Total electronic energy= -3616.794810 E_0
Sum of electronic and zero-point Energies= -3616.163228 $E_0 + E_{ZPE}$
Sum of electronic and thermal Energies= -3616.115765 $E_0 + E_{tot}$
Sum of electronic and thermal Enthalpies= -3616.114757 $E_0 + H_{corr}$
Sum of electronic and thermal Free Energies= -3616.248028 $E_0 + G_{corr}$
Zero-point correction (*unscaled*) = 0.631582
Number of imaginary vibrational frequencies = 1; 179i

4-S

Charge 1; multiplicity 1

Ni	0.49704800	-0.21106400	0.91120400
C	3.19010500	0.75423800	0.90173000
P	-1.24667000	-1.51106000	1.24967000
P	-0.75457200	1.11496800	-0.27284200
C	3.98894200	1.97607000	0.45075800
C	-2.66190800	-0.84238600	0.27366900
C	-2.38826000	0.30313600	-0.53461500
C	4.94098500	2.54632500	1.31384800
C	3.82311800	2.53486900	-0.82710200
C	5.71187100	3.64220100	0.91040700
H	5.07621800	2.12956900	2.31690200
C	4.59268800	3.63259300	-1.23319000
H	3.08206500	2.10854400	-1.50619700
C	5.54043400	4.19004700	-0.36707300
H	6.44386400	4.07410500	1.59854400
H	4.44984400	4.05458000	-2.23201800
H	6.13892700	5.04867300	-0.68311900
H	3.06015100	0.85003100	1.99604500
C	3.98566600	-0.55433600	0.67360400
H	3.43452800	-1.37800200	1.15730100
H	4.95963000	-0.48530900	1.18603700
N	-3.84803000	-1.41803800	0.28921200
N	-3.27643200	0.79489200	-1.37586000
C	-4.79887500	-0.91116100	-0.54310800
C	-6.09660400	-1.48742700	-0.56863300
H	-6.31093700	-2.31980600	0.10458700
C	-4.49680100	0.19267000	-1.41631600
C	-5.49277100	0.67218000	-2.30774600
C	-7.04654200	-0.99325600	-1.44010500
C	-6.74268000	0.08591300	-2.31462000
H	-5.24436900	1.50647900	-2.96673500
H	-7.51322700	0.45416300	-2.99591100
H	-8.04543200	-1.43493300	-1.46453400
O	1.94041600	0.71604900	0.24279900
C	-0.91731700	-3.23977600	0.54769500
C	-2.08628100	-4.18264600	0.87571900
C	-0.74016400	-3.09647700	-0.97352300
C	0.38055100	-3.77229100	1.18251500
H	-2.17040600	-4.36685800	1.95774000
H	-3.04720200	-3.79304700	0.50914300
H	-1.89789800	-5.15262200	0.38441100
H	0.05434800	-2.37346000	-1.22223400
H	-0.45106000	-4.07416600	-1.39383700
H	-1.67090000	-2.77939600	-1.46993400
H	0.51764800	-4.82079100	0.86891200
H	1.26463900	-3.20740300	0.84892100
H	0.34270300	-3.75556600	2.28348200
C	-1.88438800	-1.70590000	2.95110700
H	-2.84432500	-2.24244400	2.93748400

H	-1.15046000	-2.26287200	3.55212800
H	-2.02722700	-0.70737700	3.38907200
C	-1.15393500	2.71840400	0.65586700
C	-1.95466500	3.66121600	-0.25841600
C	-1.97197600	2.34575700	1.90357400
C	0.16550400	3.38765400	1.07504500
H	-1.35897000	3.99895700	-1.12003600
H	-2.87885200	3.19492300	-0.63042000
H	-2.23400700	4.55399700	0.32662900
H	-1.42996300	1.62985900	2.54293900
H	-2.15157700	3.25869000	2.49542600
H	-2.95298300	1.91608400	1.64636300
H	-0.07493600	4.34727600	1.56298400
H	0.72555000	2.77019000	1.79264000
H	0.81599000	3.60148100	0.21358800
C	-0.15225200	1.54356800	-1.93845400
H	-0.98487900	1.95738200	-2.52610700
H	0.65832800	2.28037600	-1.86050100
H	0.22501000	0.63232300	-2.42434000
C	4.18681200	-0.90600000	-0.80076800
H	4.95646000	-0.28832300	-1.28452100
H	3.24090600	-0.73066500	-1.34601200
C	4.50914600	-2.36195700	-1.01401000
O	4.05308800	-3.28495000	-0.35786600
O	5.33996500	-2.56365500	-2.04756000
H	5.46788300	-3.52873100	-2.15758000
H	1.56064500	-1.15869400	1.87535600
H	1.09799900	-0.77685900	2.39819500

DFT TPSSh; def2-TZVPP basis set on Ni, def2-SVP on the other atoms; 2,2,2-trifluoroethanol, SMD model
Total electronic energy= -3616.803639 E_0
Sum of electronic and zero-point Energies= -3616.167593 $E_0 + E_{ZPE}$
Sum of electronic and thermal Energies= -3616.121136 $E_0 + E_{tot}$
Sum of electronic and thermal Enthalpies= -3616.120129 $E_0 + H_{corr}$
Sum of electronic and thermal Free Energies= -3616.250923 $E_0 + G_{corr}$
Zero-point correction (*unscaled*) = 0.636046
Number of imaginary vibrational frequencies = 0

TS3-S

Charge 1; multiplicity 1

Ni	0.44407900	-0.56133200	0.54699800
C	3.19386400	0.70124500	0.85202100
P	-1.32090900	-1.76595300	0.90037500
P	-0.81019000	1.04676300	-0.29878000
C	3.71814100	1.88414800	0.04709100
C	-2.78003600	-0.86644400	0.20783600
C	-2.51436100	0.36226200	-0.47330500
C	4.24000500	3.00390800	0.71817700
C	3.72809400	1.87473300	-1.35713800
C	4.76387200	4.08654800	0.00383600
H	4.23140300	3.03002800	1.81211500
C	4.24964100	2.95973400	-2.07378100
H	3.31959000	1.01413400	-1.89089100
C	4.77028900	4.06821100	-1.39682700
H	5.16236300	4.95064400	0.54247600
H	4.24886900	2.93681500	-3.16710700
H	5.17594500	4.91517700	-1.95649100
H	2.70835300	1.10445000	1.75636300
C	4.33004100	-0.22428800	1.33830200
H	3.88880200	-0.98825400	2.00051900
H	5.02286600	0.36592100	1.96010900
N	-3.99805900	-1.36163600	0.30353000
N	-3.45715600	1.01318800	-1.12721800
C	-4.99979000	-0.68897000	-0.32677000
C	-6.33320900	-1.17221400	-0.25325600
H	-6.53146300	-2.07024000	0.33528300
C	-4.71683700	0.49828200	-1.08878700
C	-5.77062000	1.15274400	-1.78026700
C	-7.33806000	-0.50799000	-0.92791200
C	-7.05531100	0.65398600	-1.69708600
H	-5.53704700	2.04859900	-2.35907700
H	-7.86995600	1.15680800	-2.22338700
H	-8.36496000	-0.87719400	-0.87657300
O	2.25934400	-0.03984300	0.06856200
C	-1.24444200	-3.40974400	-0.03884900
C	-2.47582800	-4.27129400	0.29036400
C	-1.19400800	-3.07965900	-1.54058400
C	0.03395200	-4.15549000	0.38248200
H	-2.48383200	-4.58409800	1.34580000
H	-3.41807900	-3.74916600	0.07128500
H	-2.43454200	-5.18341000	-0.32894500
H	-0.35242200	-2.40827500	-1.77925100
H	-1.05356100	-4.01438900	-2.10855600
H	-2.12660800	-2.61021500	-1.89158800
H	0.02066200	-5.15568200	-0.08212500
H	0.94276500	-3.63702500	0.04358500
H	0.09441400	-4.29509100	1.47374400
C	-1.75917700	-2.12510100	2.63678300
H	-2.76632700	-2.56436900	2.68577400

H	-1.02131000	-2.82204800	3.06029300
H	-1.73982300	-1.18545700	3.20769600
C	-1.02956800	2.57240400	0.80123800
C	-1.83946500	3.65697100	0.07423500
C	-1.74968800	2.12331200	2.08394400
C	0.37315000	3.10101000	1.14272800
H	-1.30410200	4.04566100	-0.80557100
H	-2.82608600	3.29270200	-0.24778200
H	-1.99557600	4.49939300	0.76983800
H	-1.20712500	1.30786800	2.58990000
H	-1.80191500	2.97641500	2.78101200
H	-2.78061800	1.78953800	1.88609600
H	0.26894900	4.01932900	1.74492500
H	0.94593000	2.37142700	1.73433700
H	0.94881900	3.35588200	0.23986400
C	-0.33520600	1.61101500	-1.96973900
H	-1.13883200	2.21748000	-2.41219100
H	0.59077800	2.20036700	-1.90257000
H	-0.15311000	0.72582700	-2.59679800
C	5.09787500	-0.91300400	0.21514400
H	5.61072600	-0.18826400	-0.43949800
H	4.41914600	-1.48195500	-0.44283500
C	6.14013000	-1.87612500	0.72133600
O	6.35220100	-2.14054800	1.89175100
O	6.83643300	-2.43721200	-0.28164500
H	7.49266600	-3.05265800	0.10442900
H	1.93122700	-1.17255700	0.78134700
H	1.28205400	-1.74574800	1.21504600

DFT TPSSh; def2-TZVPP basis set on Ni, def2-SVP on the other atoms; 2,2,2-trifluoroethanol, SMD model
Total electronic energy= -3616.784392 E_0
Sum of electronic and zero-point Energies= -3616.151071 $E_0 + E_{ZPE}$
Sum of electronic and thermal Energies= -3616.104845 $E_0 + E_{tot}$
Sum of electronic and thermal Enthalpies= -3616.103838 $E_0 + H_{corr}$
Sum of electronic and thermal Free Energies= -3616.237105 $E_0 + G_{corr}$
Zero-point correction (*unscaled*) = 0.633321
Number of imaginary vibrational frequencies = 1; 1098i

5-S

Charge 1; multiplicity 1

Ni	0.45803800	-0.64334500	0.77026700
C	3.01992300	1.00680400	0.48225700
P	-1.22906200	-1.82954500	0.33105300
P	-0.70659900	1.25492000	0.48781100
C	3.29755500	0.61099000	-0.95806200
C	-2.67213100	-0.70175000	0.01845000
C	-2.39467800	0.70233700	-0.03720800
C	3.42597300	1.62222100	-1.92597700
C	3.46437600	-0.72698600	-1.35002800
C	3.71611000	1.30318600	-3.25640600
H	3.29416200	2.66842900	-1.63549000
C	3.75091700	-1.04693300	-2.68328100
H	3.36994700	-1.53093500	-0.61583200
C	3.87811100	-0.03393400	-3.63976400
H	3.80899300	2.10145100	-3.99746300
H	3.87573000	-2.09370700	-2.97250000
H	4.10019200	-0.28459500	-4.68022500
H	2.35104900	1.87636200	0.48203100
C	4.27206900	1.39456000	1.28181900
H	3.94972500	1.71807500	2.28453500
H	4.71003000	2.27687900	0.78932300
N	-3.88553000	-1.17994300	-0.17677000
N	-3.31827100	1.57512300	-0.39434300
C	-4.86829200	-0.29463100	-0.50205900
C	-6.19434100	-0.75409300	-0.72011000
H	-6.40578600	-1.81785800	-0.59312800
C	-4.56900600	1.10265700	-0.65324800
C	-5.59739300	1.99904900	-1.04866200
C	-7.17557200	0.14269600	-1.09416000
C	-6.87518400	1.52183000	-1.26374600
H	-5.35065000	3.05632800	-1.16580400
H	-7.67008500	2.20843700	-1.56432100
H	-8.19634800	-0.20673100	-1.26622100
O	2.26673600	-0.01010100	1.19644700
C	-1.04602800	-2.83070700	-1.26921500
C	-2.29080500	-3.69904600	-1.51855800
C	-0.84803700	-1.82645400	-2.41797700
C	0.19130300	-3.73588200	-1.14097100
H	-2.41061400	-4.47270500	-0.74436300
H	-3.21298600	-3.10209900	-1.56077100
H	-2.16902200	-4.21273400	-2.48752700
H	0.01869100	-1.16977400	-2.23554700
H	-0.66298700	-2.37911300	-3.35449000
H	-1.73821300	-1.19538600	-2.57156500
H	0.25314800	-4.37325000	-2.03948700
H	1.12098700	-3.15210000	-1.07184300
H	0.12919900	-4.39979100	-0.26382300
C	-1.84568400	-2.97002200	1.62169800
H	-2.85369300	-3.32611800	1.36466300

H	-1.15540700	-3.82078000	1.71744200
H	-1.88247800	-2.42837100	2.57846700
C	-1.01113400	2.15865600	2.13069600
C	-1.89821400	3.39909200	1.95604800
C	-1.67037200	1.15139600	3.08871500
C	0.36696500	2.57231300	2.67742800
H	-1.42068500	4.15749200	1.31634600
H	-2.87947700	3.14998500	1.52627000
H	-2.06542500	3.85703900	2.94651400
H	-1.05429300	0.24397500	3.20568400
H	-1.78250800	1.61523200	4.08344200
H	-2.67300400	0.84949100	2.74514500
H	0.22733800	3.09570400	3.63877800
H	1.01285900	1.69934500	2.85766400
H	0.88855900	3.26317200	1.99626300
C	-0.30378400	2.54787900	-0.75190400
H	-1.17266900	3.20257800	-0.91389400
H	0.55189500	3.14484000	-0.40474800
H	-0.03728900	2.05432200	-1.69845200
C	5.33728800	0.30845400	1.40325400
H	5.66908400	-0.04848700	0.41277100
H	4.97346300	-0.58350200	1.94074000
C	6.57066200	0.79088500	2.12569800
O	6.78316300	1.93870100	2.47169400
O	7.44283600	-0.20576900	2.34310900
H	8.22941300	0.16340100	2.79410000
H	2.83166100	-0.77318000	1.40933800
H	0.89296400	-2.04756200	1.02045700

DFT TPSSh; def2-TZVPP basis set on Ni, def2-SVP on the other atoms; 2,2,2-trifluoroethanol, SMD model
Total electronic energy= -3616.820860 E_0
Sum of electronic and zero-point Energies= -3616.182404 $E_0 + E_{ZPE}$
Sum of electronic and thermal Energies= -3616.135917 $E_0 + E_{tot}$
Sum of electronic and thermal Enthalpies= -3616.134909 $E_0 + H_{corr}$
Sum of electronic and thermal Free Energies= -3616.267408 $E_0 + G_{corr}$
Zero-point correction (*unscaled*) = 0.638456
Number of imaginary vibrational frequencies = 0

6-S

Charge 0; multiplicity 1

C	3.01029100	0.76030700	0.53730300
C	3.31125400	0.46908000	-0.93347100
C	3.92799500	1.43519800	-1.75067000
C	2.94085400	-0.75556800	-1.51175400
C	4.17559400	1.17696300	-3.10269500
H	4.21419100	2.40368100	-1.33206800
C	3.19138600	-1.01686800	-2.86494000
H	2.44407800	-1.50813500	-0.89564800
C	3.81130500	-0.05276800	-3.66595500
H	4.65587900	1.94133500	-3.71976800
H	2.89596200	-1.97876200	-3.29314800
H	4.00712400	-0.25478100	-4.72234500
H	2.19445900	1.50240200	0.56665100
C	4.19248100	1.36857500	1.31409000
H	3.83600600	1.59735200	2.33132600
H	4.47365000	2.33048200	0.85776500
O	2.47601200	-0.37379700	1.21347300
C	5.42009000	0.46578400	1.39467300
H	5.80241800	0.20598900	0.39208800
H	5.19345200	-0.49450300	1.88936500
C	6.56156600	1.09299100	2.15343900
O	6.56468200	2.21429100	2.62910000
O	7.61501100	0.26479900	2.25134300
H	8.32563600	0.72570000	2.74224700
H	3.14779000	-1.07747900	1.22168700

DFT TPSSh; def2-TZVPP basis set on Ni, def2-SVP on the other atoms; 2,2,2-trifluoroethanol, SMD model
Total electronic energy= -613.581781 E_0
Sum of electronic and zero-point Energies= -613.377358 $E_0 + E_{ZPE}$
Sum of electronic and thermal Energies= -613.363586 $E_0 + E_{tot}$
Sum of electronic and thermal Enthalpies= -613.362578 $E_0 + H_{corr}$
Sum of electronic and thermal Free Energies= -613.421859 $E_0 + G_{corr}$
Zero-point correction (*unscaled*) = 0.204423
Number of imaginary vibrational frequencies = 0

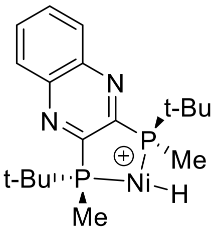

1 (M11-L)

Charge 1; multiplicity 1

Ni	0.61145800	-0.60557300	0.27485200
P	-0.99145100	-1.39233500	1.35928200
P	-0.73790800	0.31312400	-1.22138900
C	-2.51799700	-0.63436500	0.68839000
C	-2.41659300	0.04757800	-0.55578600
H	1.35555100	-1.16764500	1.41237100
N	-3.64814700	-0.75373700	1.32056900
N	-3.46541200	0.50515400	-1.17548100
C	-4.73857100	-0.24718100	0.72619500
C	-5.99052800	-0.33589600	1.36439300
H	-6.03797500	-0.80104000	2.35894500
C	-4.65017100	0.36132200	-0.56350000
C	-5.82168000	0.83497300	-1.18482500
C	-7.10975000	0.14284200	0.73718300
C	-7.02574700	0.72515600	-0.54208000
H	-5.73586400	1.29369000	-2.17980100
H	-7.94020600	1.09772700	-1.02488700
H	-8.08828900	0.07014500	1.23248300
C	-1.19543800	-3.22767300	1.07280000
C	-2.34664200	-3.76887700	1.89449400
C	-1.45117900	-3.42781200	-0.40795500
C	0.09830600	-3.90765500	1.47517400
H	-2.16756800	-3.68356300	2.98214900
H	-3.30894000	-3.27698300	1.66549200
H	-2.47163800	-4.84711200	1.67389000
H	-0.64267900	-2.99465600	-1.03087600
H	-1.49493400	-4.50987300	-0.63790400
H	-2.41272500	-2.98916100	-0.73861000
H	-0.01972200	-5.00430500	1.37679600
H	0.94703100	-3.60607900	0.83394500
H	0.37927900	-3.70570900	2.52646600
C	-1.08487500	-1.09013100	3.11951200
H	-2.08789100	-1.33592400	3.51056300
H	-0.32555100	-1.68956500	3.65106900
H	-0.88074100	-0.02356700	3.31878900
C	-0.47190400	2.15737400	-1.19716600
C	-1.31938400	2.88839400	-2.21246300
C	-0.78341700	2.62848400	0.20992200
C	1.00431700	2.34761400	-1.49506200
H	-1.08099800	2.59603500	-3.25187600
H	-2.40154300	2.73268400	-2.05333100
H	-1.12996400	3.97738900	-2.13515000

H	-0.21754000	2.05864200	0.97541900
H	-0.50299200	3.69368800	0.32357800
H	-1.86001300	2.54901300	0.45526200
H	1.26117300	3.42475300	-1.47560900
H	1.64847600	1.84513600	-0.74458500
H	1.28870800	1.96468300	-2.49388100
C	-0.85715400	-0.20225000	-2.93527700
H	-1.72274500	0.26546200	-3.43787900
H	0.06507400	0.06455100	-3.48116800
H	-0.97493200	-1.29945300	-2.98253600

DFT M11-L; def2-TZVPP basis set on Ni, def2-SVP on the other atoms; 2,2,2-trifluoroethanol, SMD model
Total electronic energy= -3002.690325 E_0
Sum of electronic and zero-point Energies= -3002.263539 $E_0 + E_{ZPE}$
Sum of electronic and thermal Energies= -3002.233023 $E_0 + E_{tot}$
Sum of electronic and thermal Enthalpies= -3002.232015 $E_0 + H_{corr}$
Sum of electronic and thermal Free Energies= -3002.324339 $E_0 + G_{corr}$
Zero-point correction (*unscaled*) = 0.426787
Number of imaginary vibrational frequencies = 0

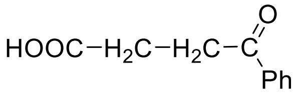

4-oxo-4-phenylbutanoic acid (M11-L)

Charge 0; multiplicity 1

C	3.51232000	0.10140100	-0.33570700
O	2.49601600	-0.53106800	-0.47533900
C	3.68022300	1.03544200	0.81945500
C	2.47762800	1.05672600	1.71451400
H	3.91058600	2.04636100	0.42538300
H	4.59465200	0.74562200	1.37680000
H	1.56861200	1.36077500	1.15822700
H	2.23130000	0.04289700	2.08531300
C	2.55234500	1.93871000	2.91034000
O	1.69790800	1.99839300	3.74672800
O	3.64752600	2.65471400	2.96224300
H	3.62070500	3.19734800	3.76237000
C	4.62464400	-0.03675600	-1.31235900
C	5.81764100	0.68099200	-1.21026300
C	4.45474400	-0.92765000	-2.37377300
C	6.81787900	0.50861400	-2.15336500
H	5.97683800	1.38869300	-0.38434600
C	5.45445200	-1.09931900	-3.31351800
H	3.51228900	-1.48931900	-2.44604200
C	6.63821100	-0.38025200	-3.20405500
H	7.75458000	1.07759000	-2.06741500
H	5.31277800	-1.80405600	-4.14551900
H	7.43455600	-0.51507500	-3.95059100

DFT M11-L; def2-TZVPP basis set on Ni, def2-SVP on the other atoms; 2,2,2-trifluoroethanol, SMD model
Total electronic energy= -612.0417649 E_0
Sum of electronic and zero-point Energies= -611.861770 $E_0 + E_{ZPE}$
Sum of electronic and thermal Energies= -611.848438 $E_0 + E_{tot}$
Sum of electronic and thermal Enthalpies= -611.847430 $E_0 + H_{corr}$
Sum of electronic and thermal Free Energies= -611.905520 $E_0 + G_{corr}$
Zero-point correction (*unscaled*) = 0.179995
Number of imaginary vibrational frequencies = 0

1-R (M11-L)

Charge 1; multiplicity 1

Ni	0.66578800	-0.12006500	0.67996500
C	3.34482600	0.64476100	0.10301800
P	-0.92112800	-1.41729200	1.19852300
P	-0.65613700	1.01643700	-0.67929600
C	-2.42580200	-0.87097900	0.30736400
C	-2.24702200	0.12345300	-0.69503400
H	1.35207900	-0.93424800	1.69564800
N	-3.58535800	-1.41383700	0.53775700
N	-3.19952300	0.44703500	-1.52115300
C	-4.59923400	-1.05686100	-0.26474800
C	-5.88041000	-1.60555700	-0.06270800
H	-6.02628100	-2.29471500	0.78095200
C	-4.38835600	-0.14959000	-1.34761300
C	-5.45583600	0.14801200	-2.21669000
C	-6.90102400	-1.28600600	-0.91787500
C	-6.68657700	-0.41231800	-2.00116300
H	-5.27277900	0.84129400	-3.04967900
H	-7.52174600	-0.17636600	-2.67574900
H	-7.90006100	-1.71803600	-0.76439200
O	2.19383600	0.90434500	0.42344100
C	-0.67359200	-3.17262200	0.58966800
C	-1.76364500	-4.08749600	1.10872500
C	-0.69693000	-3.12490700	-0.92638900
C	0.67423000	-3.66533400	1.07909100
H	-1.72771000	-4.20483500	2.20763800
H	-2.77846600	-3.75132300	0.83279200
H	-1.62382800	-5.09722400	0.67503900
H	-0.00172500	-2.36276100	-1.33265300
H	-0.38723600	-4.10443600	-1.34016800
H	-1.70565700	-2.90981400	-1.32895200
H	0.79243900	-4.73177200	0.80399000
H	1.51858100	-3.11021300	0.63112100
H	0.77585200	-3.60205200	2.17971000
C	-1.42417600	-1.50833400	2.91610600
H	-2.39769300	-2.01792400	3.02632900
H	-0.66163600	-2.04559900	3.50723100
H	-1.51410600	-0.48707200	3.32688800
C	-1.12588000	2.69496800	0.00798500
C	-1.96763200	3.48871000	-0.96589800
C	-1.88403400	2.45350400	1.29896100
C	0.16888400	3.42824900	0.30032900
H	-1.41221300	3.75764100	-1.88335700
H	-2.89033400	2.96094300	-1.26735600
H	-2.27653400	4.44028100	-0.48955900
H	-1.33041600	1.78473200	1.99000700
H	-2.03838800	3.41272000	1.83042500
H	-2.88613200	2.01402400	1.12919400
H	-0.05329600	4.46230200	0.62916800
H	0.74646400	2.94039600	1.10708400

H	0.82272000	3.50523700	-0.59056500
C	-0.24296700	1.25120700	-2.41017000
H	-1.08113100	1.68835200	-2.98172500
H	0.63886100	1.91041400	-2.50427400
H	0.01353300	0.27627700	-2.86268100
C	4.40575500	1.64816500	0.35549700
C	3.89433200	2.94764500	0.90122900
H	4.98891900	1.78711800	-0.57722900
H	5.13315100	1.18334000	1.05359600
H	3.17161600	3.42482500	0.21150500
H	3.31906500	2.79264500	1.83546100
C	4.92509900	3.97661500	1.21217000
O	4.66382700	5.08761400	1.57050800
O	6.15069400	3.54371800	1.06230400
H	6.76081800	4.25881900	1.28999000
C	3.68857300	-0.63379700	-0.53365100
C	4.94679400	-1.22190600	-0.38064500
C	2.72714000	-1.26885400	-1.32423600
C	5.21785100	-2.44134800	-0.97628600
H	5.71589800	-0.74213600	0.24047200
C	3.01236500	-2.47460300	-1.94001300
H	1.75464200	-0.77908000	-1.49414600
C	4.25413900	-3.06729900	-1.75589100
H	6.19981800	-2.91260200	-0.83030200
H	2.25865300	-2.95800700	-2.57824300
H	4.47884700	-4.03084000	-2.23572000

DFT M11-L; def2-TZVPP basis set on Ni, def2-SVP on the other atoms; 2,2,2-trifluoroethanol, SMD model
Total electronic energy= -3614.772088 E_0
Sum of electronic and zero-point Energies= -3614.163002 $E_0 + E_{ZPE}$
Sum of electronic and thermal Energies= -3614.117843 $E_0 + E_{tot}$
Sum of electronic and thermal Enthalpies= -3614.116836 $E_0 + H_{corr}$
Sum of electronic and thermal Free Energies= -3614.243335 $E_0 + G_{corr}$
Zero-point correction (*unscaled*) = 0.609086
Number of imaginary vibrational frequencies = 0

TS1-R (M11-L)

Charge 1; multiplicity 1

Ni	0.86792400	0.30406200	0.13414400
C	3.10246000	0.50917800	-0.13405200
P	-0.50852300	-1.02571000	1.05444500
P	-0.71257000	1.15872000	-1.12403300
C	-2.17189400	-0.62904100	0.40505200
C	-2.23512800	0.23827300	-0.72073200
H	1.80832100	-0.32592100	1.09209800
N	-3.23479900	-1.18417700	0.90865600
N	-3.33997400	0.42564800	-1.38232500
C	-4.39822300	-0.95888900	0.28059000
C	-5.58727500	-1.52202300	0.78261700
H	-5.53597600	-2.11013500	1.70962200
C	-4.44206200	-0.18051500	-0.91681500
C	-5.66719600	-0.02244600	-1.59303400
C	-6.76487200	-1.33890800	0.10820700
C	-6.80374100	-0.59293800	-1.08544700
H	-5.68032900	0.57258400	-2.51691600
H	-7.76156800	-0.46535700	-1.60941000
H	-7.69279800	-1.78171700	0.49680700
O	2.24276300	1.23301800	-0.69191300
C	-0.22567000	-2.78870200	0.49263700
C	-1.17348900	-3.74035100	1.19125900
C	-0.44211900	-2.81832100	-1.00864300
C	1.20838300	-3.15713500	0.81829000
H	-0.99499800	-3.78841000	2.28141400
H	-2.23673800	-3.48966900	1.02925800
H	-1.01567600	-4.76286000	0.79575600
H	0.16817500	-2.05378100	-1.53314600
H	-0.14454700	-3.80552400	-1.41251200
H	-1.50133300	-2.65974300	-1.29035800
H	1.37725600	-4.22402000	0.57345700
H	1.93381300	-2.56391400	0.23218200
H	1.45003700	-3.02973200	1.89120400
C	-0.69256000	-1.03735400	2.83415500
H	-1.59239900	-1.59963700	3.13988400
H	0.19850800	-1.48947600	3.30435600
H	-0.78284300	-0.00026300	3.20202100
C	-1.12968500	2.93098900	-0.70133600
C	-2.13750500	3.51429800	-1.66705600
C	-1.67348500	2.93405700	0.71413000
C	0.16572500	3.71772100	-0.75834500
H	-1.73869000	3.59392900	-2.69528300
H	-3.07782100	2.93632600	-1.70875400
H	-2.39808700	4.54182100	-1.34501800
H	-0.99001700	2.42642800	1.42537300
H	-1.79293100	3.97609800	1.06908900
H	-2.66643000	2.45083900	0.79229200
H	-0.04656300	4.79203800	-0.59399600
H	0.87948200	3.39859300	0.02325200

H	0.67611800	3.63419500	-1.73703200
C	-0.54809200	1.04491400	-2.90536600
H	-1.48093800	1.33030700	-3.42392900
H	0.27133300	1.69852100	-3.25480300
H	-0.30002300	0.00504400	-3.18483200
C	3.89987400	1.07383200	0.99509200
C	3.21084900	2.22153900	1.66966600
H	4.86256900	1.39994900	0.54371200
H	4.17732400	0.28673800	1.71840300
H	3.02056100	3.04838000	0.95649200
H	2.19983000	1.92774600	2.02280000
C	3.91689700	2.83081000	2.82974900
O	3.41171500	3.62165900	3.57226300
O	5.15618600	2.42871300	2.95104000
H	5.55075400	2.87667600	3.71199800
C	3.55993600	-0.71644900	-0.83008300
C	4.53557300	-1.56070300	-0.29794900
C	2.96142300	-1.04888200	-2.04864300
C	4.89005500	-2.72246300	-0.96423100
H	5.02427700	-1.32347700	0.65689600
C	3.31760200	-2.20990900	-2.71008300
H	2.20115900	-0.37640100	-2.47436900
C	4.27844800	-3.05307600	-2.16524400
H	5.65550200	-3.38448100	-0.53515800
H	2.84148400	-2.46336400	-3.66810500
H	4.55809700	-3.97917700	-2.68807900

DFT M11-L; def2-TZVPP basis set on Ni, def2-SVP on the other atoms; 2,2,2-trifluoroethanol, SMD model
Total electronic energy= -3614.764428 E_0
Sum of electronic and zero-point Energies= -3614.156740 $E_0 + E_{ZPE}$
Sum of electronic and thermal Energies= -3614.112105 $E_0 + E_{tot}$
Sum of electronic and thermal Enthalpies= -3614.111098 $E_0 + H_{corr}$
Sum of electronic and thermal Free Energies= -3614.236288 $E_0 + G_{corr}$
Zero-point correction (*unscaled*) = 0.607688
Number of imaginary vibrational frequencies = 1; 325i

2-R (M11-L)

Charge 1; multiplicity 1

Ni	0.79625000	0.31412300	0.05046600
C	2.89889600	0.27779400	-0.12772200
P	-0.60663500	-0.96010900	1.12120000
P	-0.79171400	1.18635900	-1.10311000
C	-2.23935700	-0.66212400	0.35854800
C	-2.28730700	0.20520600	-0.76587000
H	2.20362900	-0.28215300	0.69803200
N	-3.30657400	-1.24340700	0.82308600
N	-3.37186000	0.37541900	-1.46367100
C	-4.44969200	-1.05037100	0.15000700
C	-5.64024200	-1.64907500	0.60546700
H	-5.60568400	-2.24250700	1.52971600
C	-4.47263400	-0.26460300	-1.04411600
C	-5.67659800	-0.13382600	-1.76260400
C	-6.79717900	-1.49291000	-0.10984400
C	-6.81402300	-0.73895100	-1.29924100
H	-5.67330800	0.46765900	-2.68227700
H	-7.75587200	-0.63354500	-1.85600400
H	-7.72602200	-1.96357200	0.24200500
O	2.12212500	1.12439300	-0.80273300
C	-0.20679400	-2.75636200	0.80534900
C	-1.32773300	-3.68281900	1.22258800
C	0.06078700	-2.88075200	-0.68300800
C	1.04585700	-3.07433500	1.59843200
H	-1.61588100	-3.55988100	2.28316400
H	-2.23512900	-3.56366600	0.60537200
H	-0.98829500	-4.73042800	1.10215000
H	0.92148800	-2.26071400	-1.00607400
H	0.29722600	-3.93196300	-0.93842500
H	-0.81476300	-2.58667500	-1.29539600
H	1.40680800	-4.08521400	1.32605800
H	1.87424500	-2.36985000	1.39162700
H	0.86535200	-3.07822000	2.68954900
C	-0.88920700	-0.74196600	2.87608700
H	-1.68079500	-1.41900300	3.24436100
H	0.03961500	-0.92677100	3.44306200
H	-1.20269100	0.29975800	3.06714500
C	-1.22787500	2.91854500	-0.57020500
C	-2.25853000	3.52675200	-1.49625700
C	-1.75563100	2.83094900	0.84814500
C	0.05393200	3.72721700	-0.59951800
H	-1.87494300	3.66043900	-2.52447700
H	-3.18950800	2.93462100	-1.55184100
H	-2.53244800	4.53280500	-1.12258900
H	-1.04669600	2.31057500	1.52423800
H	-1.90252400	3.84966100	1.25573200
H	-2.73284800	2.31430300	0.91078000
H	-0.17129800	4.78399200	-0.35825100
H	0.78782800	3.36743200	0.14537300

H	0.54424200	3.71812600	-1.59131500
C	-0.55773800	1.17009900	-2.87495000
H	-1.48645200	1.44495900	-3.40644200
H	0.24377400	1.87513800	-3.15838600
H	-0.25667200	0.15597600	-3.19222100
C	3.91792400	0.95705400	0.77050500
C	3.28212100	1.99374400	1.65050500
H	4.67797700	1.41378300	0.10473100
H	4.45391600	0.20271600	1.37706900
H	2.86109500	2.82094100	1.04319900
H	2.40924900	1.58148600	2.19937700
C	4.16277500	2.64134300	2.66087800
O	3.75898400	3.27664600	3.59140500
O	5.43674400	2.46549500	2.41707600
H	5.94745900	2.92474500	3.09792000
C	3.44151300	-0.86773800	-0.95451600
C	4.11058200	-1.93853800	-0.36566800
C	3.22667700	-0.87660300	-2.32893500
C	4.55145500	-3.00171000	-1.13834700
H	4.28021700	-1.95418100	0.72213600
C	3.67712500	-1.93647900	-3.10287100
H	2.69477100	-0.03349700	-2.79331800
C	4.33595500	-3.00374800	-2.50997900
H	5.07081200	-3.84523000	-0.66053500
H	3.50646500	-1.92990800	-4.18954600
H	4.68554400	-3.84776900	-3.12226200

DFT M11-L; def2-TZVPP basis set on Ni, def2-SVP on the other atoms; 2,2,2-trifluoroethanol, SMD model
Total electronic energy= -3614.782205 E_0
Sum of electronic and zero-point Energies= -3614.170162 $E_0 + E_{ZPE}$
Sum of electronic and thermal Energies= -3614.125641 $E_0 + E_{tot}$
Sum of electronic and thermal Enthalpies= -3614.124633 $E_0 + H_{corr}$
Sum of electronic and thermal Free Energies= -3614.250596 $E_0 + G_{corr}$
Zero-point correction (*unscaled*) = 0.612043
Number of imaginary vibrational frequencies = 0

1-S (M11-L)

Charge 1; multiplicity 1

Ni	0.56935500	-0.08599600	1.23174100
C	3.24116100	0.69645900	0.73584300
P	-1.06627600	-1.41059800	1.46671100
P	-0.55140500	1.07205900	-0.28571100
C	4.21093600	1.79464000	0.71604400
C	-2.44535800	-0.81851300	0.41921400
C	-2.13396900	0.19755000	-0.52763400
C	5.42723200	1.69297500	0.03321100
C	3.89009700	2.99015900	1.37102300
C	6.29569000	2.77005400	-0.00246200
H	5.69554600	0.77021200	-0.49952000
C	4.76914400	4.05481300	1.34679200
H	2.94166400	3.06368400	1.92122200
C	5.97095100	3.94743900	0.65622600
H	7.24399600	2.68839200	-0.55156100
H	4.51889300	4.98546200	1.87521200
H	6.66822900	4.79750500	0.63467800
H	1.11678900	-0.89512800	2.33339900
C	3.60425800	-0.68549800	0.31901800
H	3.18285900	-1.34789600	1.10524600
H	4.69472100	-0.84959800	0.31314400
N	-3.62596600	-1.36062500	0.48407100
N	-2.96858900	0.54452000	-1.46415400
C	-4.52608600	-0.97710900	-0.43398400
C	-5.82479700	-1.52136300	-0.41334800
H	-6.08269200	-2.22605700	0.38967500
C	-4.17199000	-0.04862400	-1.46009500
C	-5.11463900	0.27206300	-2.45603100
C	-6.72300900	-1.17863900	-1.38864000
C	-6.36465600	-0.28567000	-2.41652700
H	-4.82118100	0.98016500	-3.24370100
H	-7.10196400	-0.03226400	-3.19135800
H	-7.73513000	-1.60726400	-1.37619600
O	2.09816200	0.94251700	1.10332200
C	-0.70359700	-3.10483300	0.74998400
C	-1.81898700	-4.07956400	1.06290900
C	-0.55506300	-2.93266700	-0.75073800
C	0.59584500	-3.60151100	1.35080600
H	-1.90300700	-4.29211400	2.14466500
H	-2.80539800	-3.73635800	0.70316700
H	-1.60695700	-5.04517900	0.56289100
H	0.13077100	-2.10195300	-1.01489100
H	-0.13878600	-3.85641400	-1.19828000
H	-1.52268100	-2.73858800	-1.25261300
H	0.80667000	-4.62329700	0.97834500
H	1.45592300	-2.96309200	1.07931900
H	0.55792600	-3.65832300	2.45581200
C	-1.79075000	-1.65023200	3.08666500
H	-2.75843200	-2.17831900	3.02178300

H	-1.10130900	-2.22735500	3.72788100
H	-1.95690900	-0.66872100	3.56484900
C	-1.09323800	2.74336000	0.36783800
C	-1.76925700	3.57243700	-0.70116300
C	-2.03948200	2.48628600	1.52503700
C	0.15081500	3.44983400	0.87151300
H	-1.07497800	3.86483500	-1.51046800
H	-2.63397200	3.06041100	-1.16075700
H	-2.14757500	4.51117700	-0.25021000
H	-1.60694400	1.78907300	2.27223700
H	-2.25410400	3.43535700	2.05377900
H	-3.01315400	2.07358500	1.19710300
H	-0.10616900	4.48397400	1.17387500
H	0.58256000	2.94300800	1.75422900
H	0.94273200	3.52541000	0.09981900
C	0.09364400	1.33381200	-1.94050200
H	-0.63837000	1.83685600	-2.59715200
H	1.01498600	1.94325700	-1.89666400
H	0.35047200	0.36019500	-2.39558500
C	2.99421900	-1.04235600	-1.01135700
H	3.45548000	-0.48134900	-1.84875000
H	1.92022200	-0.76379800	-1.04295100
C	3.07832300	-2.49581700	-1.32276000
O	3.55936500	-3.32252500	-0.60472800
O	2.54660100	-2.77146200	-2.48551700
H	2.62007300	-3.72261600	-2.64149200

DFT M11-L; def2-TZVPP basis set on Ni, def2-SVP on the other atoms; 2,2,2-trifluoroethanol, SMD model
Total electronic energy= -3614.776067 E_0
Sum of electronic and zero-point Energies= -3614.165873E_0 + E_{ZPE}
Sum of electronic and thermal Energies= -3614.121279 E_0 + E_{tot}
Sum of electronic and thermal Enthalpies= -3614.120272 E_0 + H_{corr}
Sum of electronic and thermal Free Energies= -3614.244177 E_0 + G_{corr}
Zero-point correction (*unscaled*) = 0.610194
Number of imaginary vibrational frequencies = 0

TS1-S (M11-L)

Charge 1; multiplicity 1

Ni	0.63776900	0.18894000	0.15839600
C	2.92155900	0.43831200	-0.14321100
P	-0.76330900	-1.06080700	1.14877300
P	-0.95382500	1.15278100	-1.00007300
C	3.69676100	1.11819600	0.91558300
C	-2.42439400	-0.64185900	0.49764500
C	-2.47887600	0.23064500	-0.62528800
C	4.57736700	0.42920900	1.75281300
C	3.56872300	2.50280800	1.04894400
C	5.30077000	1.11282800	2.71437900
H	4.68705200	-0.66156000	1.67221800
C	4.30228700	3.18310900	2.00424800
H	2.89871300	3.04742900	0.36905800
C	5.16494600	2.48882500	2.84213700
H	5.98276600	0.56255500	3.37781000
H	4.20453000	4.27427500	2.09526500
H	5.74441200	3.02915100	3.60470300
H	1.58316900	-0.50248900	1.06741200
C	3.44205300	-0.82683000	-0.75247300
H	2.61581400	-1.28713800	-1.32698600
H	3.74411700	-1.55793700	0.01925600
N	-3.49611300	-1.17414100	1.00656700
N	-3.58325800	0.44329400	-1.27990600
C	-4.65735900	-0.93084600	0.38099300
C	-5.85367300	-1.47547500	0.88634700
H	-5.80875000	-2.06541900	1.81254400
C	-4.69263400	-0.15043000	-0.81526000
C	-5.91719800	0.02723900	-1.48755000
C	-7.03031300	-1.27390800	0.21550400
C	-7.06095200	-0.52644200	-0.97739600
H	-5.92399600	0.62371500	-2.41057200
H	-8.01816400	-0.38359700	-1.49855300
H	-7.96375100	-1.70289600	0.60641100
O	2.01741100	1.06520700	-0.73646600
C	-0.55133900	-2.86440500	0.69643400
C	-1.51813300	-3.73628000	1.47079500
C	-0.79868900	-2.98207500	-0.79498300
C	0.87216300	-3.27001000	1.02243000
H	-1.32094800	-3.72173200	2.55866200
H	-2.57491400	-3.46017600	1.31081900
H	-1.40031200	-4.78626500	1.13844000
H	-0.15652300	-2.29263800	-1.38039700
H	-0.56562200	-4.00970400	-1.13482500
H	-1.85241600	-2.78235200	-1.07026300
H	0.98652400	-4.35951000	0.86088400
H	1.61268000	-2.76353500	0.37694900
H	1.14366200	-3.06949500	2.07666100
C	-0.92816400	-0.96264300	2.92896500
H	-1.84866700	-1.46661000	3.27305200

H	-0.05372500	-1.42806100	3.41733900
H	-0.96851500	0.09584500	3.24062700
C	-1.35186800	2.88436600	-0.41447500
C	-2.32078200	3.57478800	-1.34841200
C	-1.93748300	2.77227700	0.97976200
C	-0.03754700	3.63815200	-0.35676900
H	-1.88860100	3.75193700	-2.35048100
H	-3.26441100	3.01356800	-1.47670800
H	-2.58508200	4.56740200	-0.93319100
H	-1.29998300	2.16431300	1.65449100
H	-2.01937700	3.77885300	1.43391400
H	-2.95390500	2.33372400	0.98370100
H	-0.22574800	4.70047500	-0.10658600
H	0.63044300	3.22750200	0.42374400
H	0.51115300	3.62220600	-1.31830800
C	-0.83025500	1.20005100	-2.78662100
H	-1.78304500	1.49780900	-3.25936300
H	-0.03930600	1.90833800	-3.09188100
H	-0.54850800	0.19955200	-3.15953200
C	4.60490000	-0.53689000	-1.66863600
H	5.46160500	-0.07976900	-1.13316700
H	4.33745600	0.19910100	-2.45325900
C	5.10072500	-1.76677700	-2.34550700
O	4.65145100	-2.86266800	-2.17907000
O	6.09803800	-1.51208800	-3.15190100
H	6.38248800	-2.34040400	-3.56124400

DFT M11-L; def2-TZVPP basis set on Ni, def2-SVP on the other atoms; 2,2,2-trifluoroethanol, SMD model
Total electronic energy= -3614.758410E_0
Sum of electronic and zero-point Energies= -3614.150970 $E_0 + E_{ZPE}$
Sum of electronic and thermal Energies= -3614.106081 $E_0 + E_{tot}$
Sum of electronic and thermal Enthalpies= -3614.105074 $E_0 + H_{corr}$
Sum of electronic and thermal Free Energies= -3614.232762 $E_0 + G_{corr}$
Zero-point correction (*unscaled*) = 0.607440
Number of imaginary vibrational frequencies = 1; 248i

2-S (M11-L)

Charge 1; multiplicity 1

Ni	0.60764200	0.07826600	0.02793700
C	2.71735800	0.12200700	-0.02017300
P	-0.82309100	-1.12005700	1.14551700
P	-0.97314000	1.11946600	-0.98496800
C	3.50471500	0.95145600	0.97068300
C	-2.47210300	-0.67101100	0.50028700
C	-2.51891300	0.24444800	-0.58582600
C	4.02110200	0.38296500	2.13222300
C	3.75120400	2.29414100	0.70241000
C	4.76687300	1.14700500	3.01599900
H	3.82453500	-0.67826300	2.35567600
C	4.50414800	3.05653200	1.58446400
H	3.34774000	2.74277100	-0.21748800
C	5.01149400	2.48651600	2.74287700
H	5.16233500	0.69113400	3.93542600
H	4.69788000	4.11614400	1.36155000
H	5.60392800	3.09292800	3.44336500
H	2.00392600	-0.54138100	0.69213700
C	3.54299500	-0.94608100	-0.72336300
H	2.84997800	-1.59072700	-1.29978900
H	4.03968300	-1.60374700	0.01712600
N	-3.54911600	-1.21278100	0.98834000
N	-3.62348800	0.51382800	-1.21763300
C	-4.70862800	-0.92546800	0.37985500
C	-5.90910400	-1.48241600	0.86117800
H	-5.86734500	-2.12393300	1.75249200
C	-4.74038100	-0.07952900	-0.77219100
C	-5.96638800	0.15435300	-1.42401400
C	-7.08634500	-1.22719400	0.21035200
C	-7.11415900	-0.41196600	-0.93764500
H	-5.97051700	0.80090300	-2.31262800
H	-8.07251000	-0.22739000	-1.44325800
H	-8.02328700	-1.66527000	0.58220300
O	1.94864500	0.83219300	-0.85051800
C	-0.62131700	-2.93389500	0.76306300
C	-1.55169800	-3.78775700	1.59698800
C	-0.90159800	-3.10879200	-0.71641000
C	0.82213200	-3.28502900	1.06867300
H	-1.32889300	-3.72305300	2.67801900
H	-2.61596600	-3.53296600	1.45030900
H	-1.42459700	-4.84924000	1.30720700
H	-0.28359500	-2.43192500	-1.34118000
H	-0.66172700	-4.14452200	-1.02520500
H	-1.96418400	-2.93214700	-0.97145300
H	0.97659900	-4.37301300	0.93388600
H	1.52990600	-2.77037600	0.39097400
H	1.10775100	-3.04523700	2.11093100
C	-0.95628100	-0.93807000	2.92185900
H	-1.85838300	-1.44536400	3.30798500

H	-0.06332300	-1.35855800	3.41756400
H	-1.01781700	0.13420100	3.17885100
C	-1.22526900	2.85072100	-0.33611000
C	-2.23647600	3.59220700	-1.18412600
C	-1.70479500	2.73245600	1.09692100
C	0.11951700	3.54953400	-0.37804200
H	-1.87727400	3.76549100	-2.21519600
H	-3.21036200	3.07304700	-1.24256200
H	-2.42316500	4.58809700	-0.73684400
H	-1.03029300	2.10293800	1.71287300
H	-1.72856500	3.73505300	1.56562900
H	-2.72761500	2.31598000	1.17363900
H	-0.00910000	4.61067600	-0.08955100
H	0.84142000	3.09851900	0.32869100
H	0.57781600	3.53934700	-1.38553600
C	-0.87390100	1.18711800	-2.76717400
H	-1.81432300	1.56355000	-3.20721300
H	-0.04123200	1.84427400	-3.07416400
H	-0.67712800	0.17521800	-3.16179700
C	4.55655400	-0.33334100	-1.64655100
H	5.30054900	0.28747200	-1.10657700
H	4.08074800	0.36541700	-2.36485000
C	5.30539800	-1.34404400	-2.43837900
O	5.12142700	-2.52583300	-2.38822200
O	6.20385700	-0.79426400	-3.21641600
H	6.65062100	-1.49487500	-3.71031200

DFT M11-L; def2-TZVPP basis set on Ni, def2-SVP on the other atoms; 2,2,2-trifluoroethanol, SMD model
Total electronic energy= -3614.782263E_0
Sum of electronic and zero-point Energies= -3614.170461 $E_0 + E_{ZPE}$
Sum of electronic and thermal Energies= -3614.125746 $E_0 + E_{tot}$
Sum of electronic and thermal Enthalpies= -3614.124738 $E_0 + H_{corr}$
Sum of electronic and thermal Free Energies= -3614.251557 $E_0 + G_{corr}$
Zero-point correction (*unscaled*) = 0.611802
Number of imaginary vibrational frequencies = 0

1.3. NCI analysis

Noncovalent interaction (NCI) analysis and ploting the sign (λ_2)ρ mapped RDG isosurface was performed for **TS1-R**, **TS1-S**, **TS3-R** and **TS3-S** structures, using Multiwfn ver.3.8, with default settings as described in Multiwfn manual.[1]

Color code for NCI is:

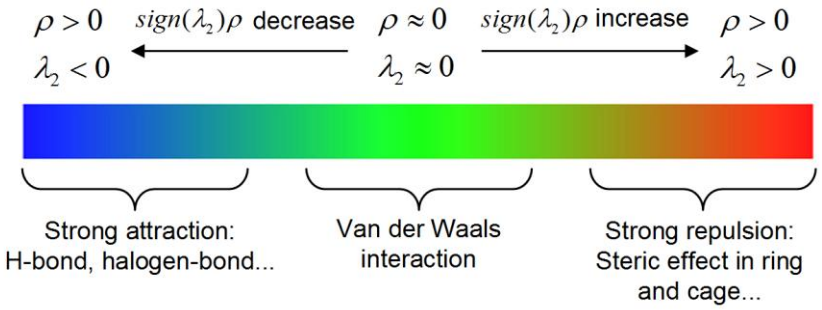

[1] Consult section 3.23.1 «Noncovalent interaction (NCI) analysis» and especially part 3 of this section in manual for Multiwfn version 3.8.

TS1-R

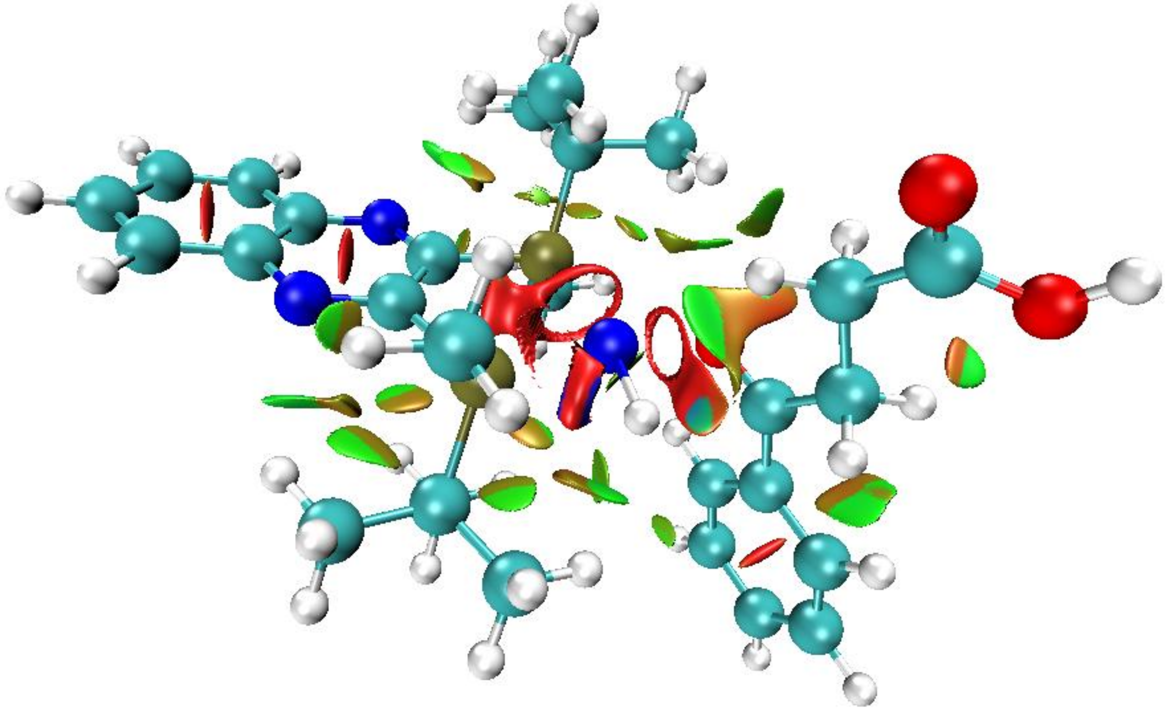

Figure S1.Visualization of NCI analysis for **TS1-R**.

TS1-S

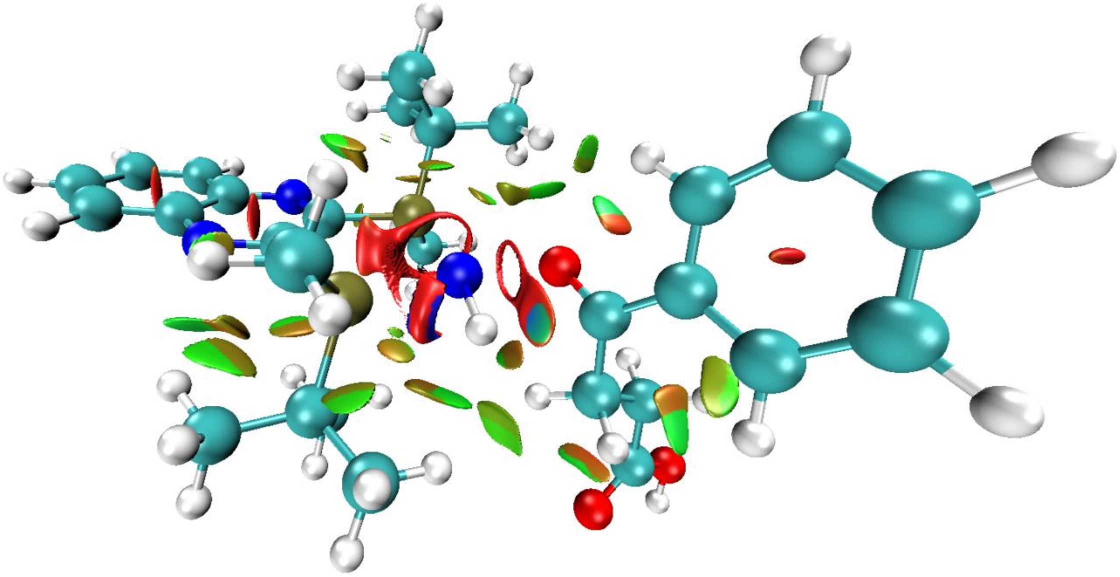

Figure S2.Visualization of NCI analysis for **TS1-S**.

TS3-R

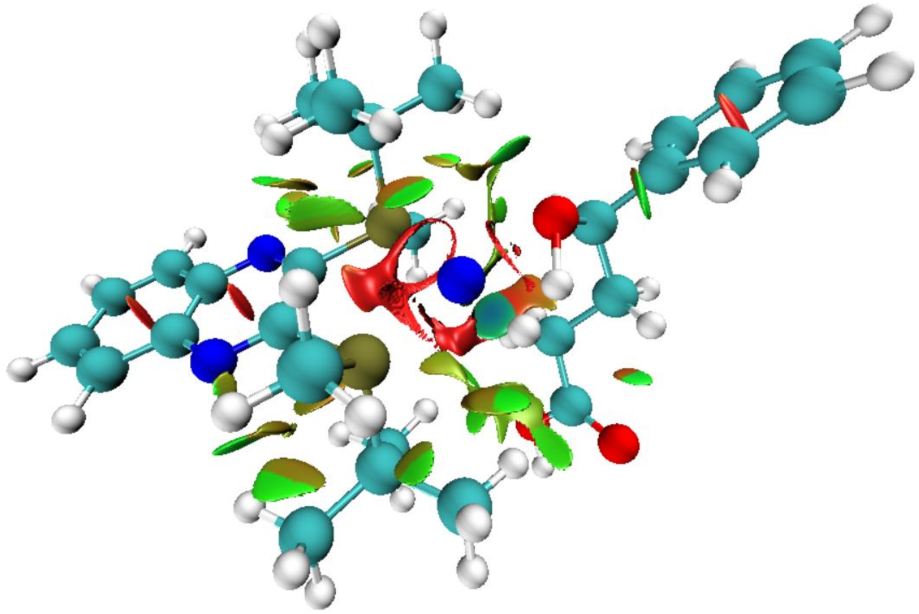

Figure S3.Visualization of NCI analysis for **TS3-R**.

TS3-S

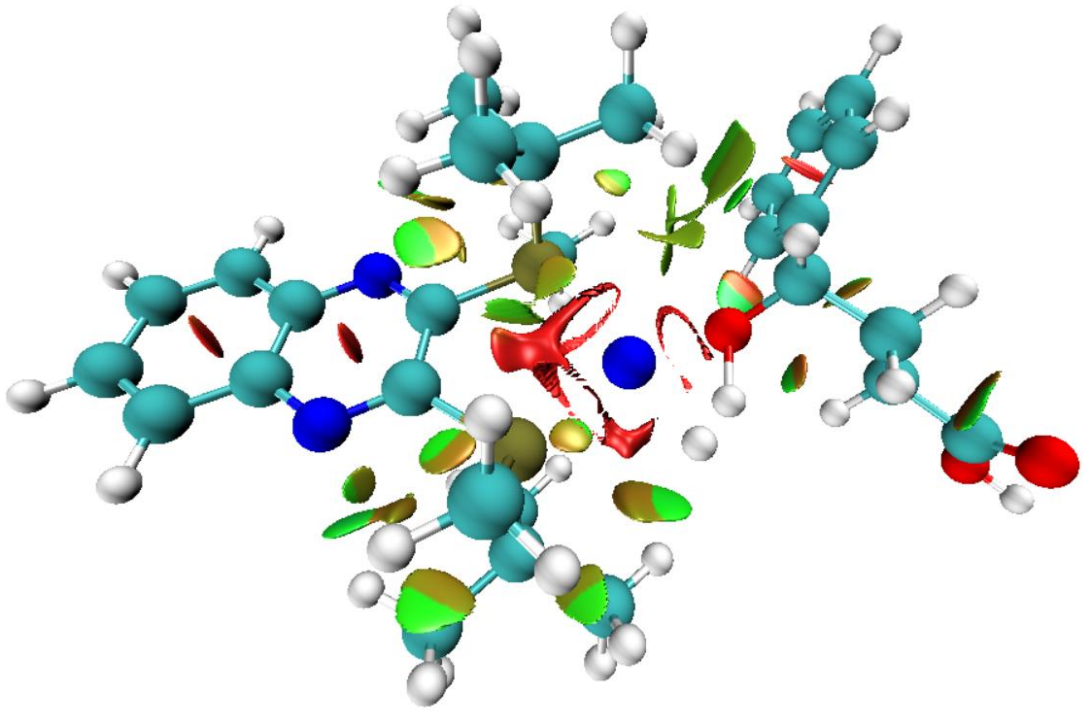

Figure S4.Visualization of NCI analysis for **TS3-S**.

1.4. SobEDAw analysis

SobEDAw analysis was done by Multiwfn ver.3.8 using sobEDA.sh script.[2] All single point calculations were performed with Gaussian 16 Rev.C01.

Analysis was performed for **TS1-R**, **TS1-S**, **TS3-R** and **TS3-S** structures, using their optimized coordinates (see section 1.2 of SI). Each structure was divided into two fragments namely catalyst and substrate. Analysis was done in gas phase using TPSSh functional; Def2TZVPP basis set on all atoms and Grimme's dispersion with Becke-Johnson damping (D3(BJ)). SobEDAw was performed with iCP=1. SobEDAw terms using parm_c/a/r defined below:

parm_c=-0.208050;parm_a=0.290588;parm_r=2.035276

[2] http://sobereva.com/soft/sobEDA_tutorial.zip

SobEDAw analysis results from original files:

TS1-R

Total interaction energy: -38.40 kcal/mol

Electrostatic (E_els): -46.62 kcal/mol

Exchange-repulsion (including scaled DFT correlation): 67.74 kcal/mol

Orbital (E_orb): -35.91 kcal/mol

Dispersion (E_disp): -23.62 kcal/mol

TS1-S

Total interaction energy: -37.64 kcal/mol

Electrostatic (E_els): -47.89 kcal/mol

Exchange-repulsion (including scaled DFT correlation): 66.88 kcal/mol

Orbital (E_orb): -35.72 kcal/mol

Dispersion (E_disp): -20.92 kcal/mol

TS3-R

Total interaction energy: -254.70 kcal/mol

Electrostatic (E_els): -273.02 kcal/mol

Exchange-repulsion (including scaled DFT correlation): 217.82 kcal/mol

Orbital (E_orb): -163.49 kcal/mol

Dispersion (E_disp): -36.02 kcal/mol

TS3-S

Total interaction energy: -250.33 kcal/mol

Electrostatic (E_els): -260.69 kcal/mol

Exchange-repulsion (including scaled DFT correlation): 193.24 kcal/mol

Orbital (E_orb): -155.20 kcal/mol

Dispersion (E_disp): -27.68 kcal/mol

1.5. ETS-NOCV analysis

The extended transition states with natural orbitals for chemical valence approach (ETS-NOCV) were calculated for catalytic particle **1**, **TS1-R** and **TS1-S**. Calculations were done as single points, using Cartesian coordinates provided above. Calculations were performed in gas phase using TPSSh functional with def2-TZVPP basis set on Ni and def2-SVP on the other atoms. Each molecule was divided into fragments (Figure S5). Fragmentation differs from SobEDAw analysis. Also for ETS-NOCV analysis atoms must be numbered in a special way, so their numbers and Cartesian coordinates are specially provided below.

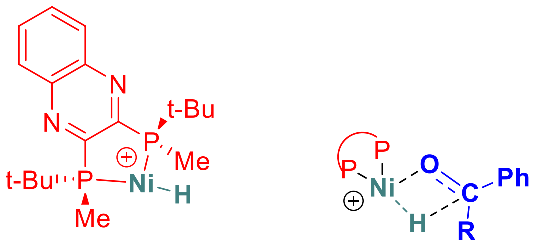

1 **TS1**

Figure S5. Fragmentation of **1** and **TS1** for ETS-NOCV.

1 (Cartesian coordinates for ETS-NOCV)

Charge 1; multiplicity 1

Ni	0.60534300	-0.64616100	0.26570300
H	1.24592500	-1.34332600	1.41961400
P	-0.98056800	-1.44280700	1.35132500
P	-0.71303900	0.37807800	-1.21334800
C	-2.52555200	-0.66363300	0.66644300
C	-2.41428100	0.09691000	-0.54337300
N	-3.67890100	-0.83412200	1.28006500
N	-3.47814900	0.59753800	-1.14280600
C	-4.78814400	-0.29623500	0.70114900
C	-6.05638300	-0.44661900	1.32166100
H	-6.11228500	-0.98098300	2.27217700
C	-4.68830100	0.40904900	-0.54739400
C	-5.86363100	0.92783900	-1.15252500
C	-7.18067900	0.07406100	0.71197700
C	-7.08446000	0.75979900	-0.52954200
H	-5.77068000	1.45680400	-2.10319900
H	-7.99081000	1.15917300	-0.99063000
H	-8.15925700	-0.04189000	1.18372700
C	-1.18487900	-3.30689900	1.10788600
C	-2.37747900	-3.82239200	1.93217500
C	-1.41270800	-3.55545400	-0.39280000
C	0.10825200	-4.00066400	1.56916300
H	-2.20648900	-3.70972000	3.01376900
H	-3.31574100	-3.31202900	1.67047800
H	-2.50327300	-4.89833300	1.72320800
H	-0.57339700	-3.17143400	-0.99542600
H	-1.48827000	-4.64139200	-0.56952200
H	-2.34524200	-3.09118000	-0.75182000
H	-0.03341000	-5.09177000	1.49015100
H	0.96766800	-3.72040800	0.94152000
H	0.35070200	-3.76884000	2.61853700
C	-1.07116000	-1.08382600	3.13837500
H	-2.08080300	-1.30829700	3.51188300
H	-0.32264700	-1.68775400	3.67092900
H	-0.85161500	-0.01689100	3.29104300
C	-0.43610400	2.24647200	-1.22601100
C	-1.34303200	2.97365700	-2.22746500
C	-0.68491500	2.75289900	0.20515300
C	1.04585600	2.43943900	-1.60138100
H	-1.13858400	2.66469600	-3.26433600
H	-2.40811900	2.79935600	-2.01473700
H	-1.15194600	4.05870300	-2.15924700
H	-0.07084000	2.20518200	0.94019300
H	-0.41455400	3.82060700	0.26576700
H	-1.74299000	2.65613800	0.49631700
H	1.28329000	3.51682500	-1.60215300
H	1.71416800	1.94553800	-0.87572500
H	1.27092900	2.04567500	-2.60560300

C	-0.84001100	-0.17095800	-2.95772200
H	-1.69621700	0.30858700	-3.45445800
H	0.09071700	0.08140500	-3.48757300
H	-0.97333000	-1.26292000	-2.97068900

Table S1. Characteristics of selected NOCV pairs of **1**.

NOCV pair	ΔE kcal/mol	Eigenvalue
1	-77.21	1.15425
2	-23.99	0.44387
3	-9.77	0.33942
4	-10.51	0.28696
5	-10.39	0.25828
Overall ΔE_{orb} = -156.48 kcal/mol		

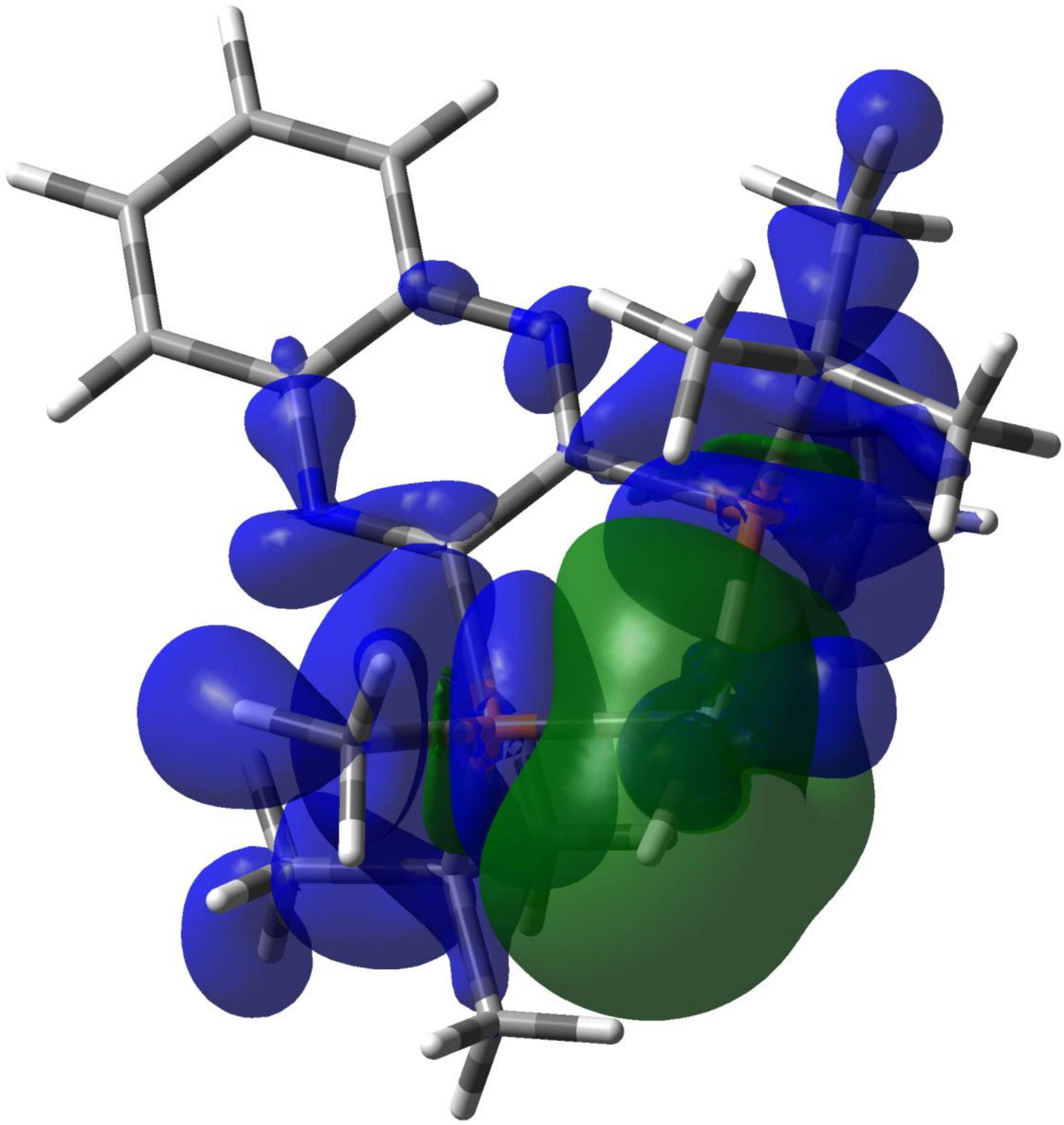

Figure S6. Visualization of sum of first and second NOCV pairs density isosurface for **1**. Isovalue = 0.004.

TS1-R (Cartesian coordinates for ETS-NOCV)

Charge 1; multiplicity 1

Ni	0.53177200	0.08674600	0.33637300
H	1.30866700	-0.71290100	1.33355700
P	-0.89453200	-1.15190600	1.27502300
P	-1.01042900	1.06604000	-0.94098600
C	-2.56270700	-0.71308700	0.57577600
C	-2.59625800	0.21323800	-0.51772100
N	-3.66129600	-1.26212100	1.05517900
N	-3.71717500	0.48104800	-1.16171100
C	-4.83802600	-0.96848000	0.43574300
C	-6.05145300	-1.53094600	0.91352800
H	-6.01724700	-2.17439700	1.79509300
C	-4.85987600	-0.11128300	-0.71726800
C	-6.09141400	0.13858700	-1.37937000
C	-7.23474800	-1.26263700	0.25425700
C	-7.25405000	-0.42964000	-0.89765700
H	-6.08910100	0.78702000	-2.25796700
H	-8.20404800	-0.23735700	-1.40190000
H	-8.17023400	-1.69592700	0.61581000
C	-0.67087300	-2.99723600	0.90434900
C	-1.75396800	-3.83544600	1.60492500
C	-0.75489200	-3.16919400	-0.62209500
C	0.71553200	-3.43457700	1.40792600
H	-1.67249400	-3.77353000	2.70121500
H	-2.76938000	-3.53268500	1.31306200
H	-1.61412500	-4.89191800	1.31919800
H	0.00241900	-2.55597200	-1.13804800
H	-0.56806000	-4.22549600	-0.87856700
H	-1.74878200	-2.89912800	-1.01397800
H	0.81669000	-4.52173400	1.24996600
H	1.52840200	-2.93406100	0.86140600
H	0.84542400	-3.23988400	2.48453300
C	-1.12111800	-0.97283000	3.08075500
H	-2.04688400	-1.47439500	3.39802000
H	-0.25567000	-1.40688900	3.60207100
H	-1.18450400	0.09878000	3.32066700
C	-1.33182700	2.90290800	-0.60262900
C	-2.38723300	3.48677300	-1.55348500
C	-1.79290700	3.02713500	0.85968100
C	0.00902900	3.63515400	-0.79511800
H	-2.04419400	3.47766400	-2.59969500
H	-3.34142400	2.94317900	-1.49428400
H	-2.57198700	4.53833000	-1.27320000
H	-1.06494400	2.57582000	1.55401900
H	-1.89065200	4.09468200	1.12000500
H	-2.77278000	2.55085700	1.02450500
H	-0.15078800	4.71618500	-0.64275100
H	0.76536400	3.29769000	-0.07112300

H	0.41284500	3.49671900	-1.81092700
C	-0.86891000	0.87555700	-2.75907500
H	-1.79263500	1.19303600	-3.26458100
H	-0.01994100	1.47599400	-3.11867300
H	-0.67678200	-0.18421100	-2.98336300
C	3.07591700	0.33239500	-0.04046300
O	2.07650600	0.94536800	-0.51088500
C	3.71380100	0.79165800	1.24841900
C	2.92252900	1.88304500	1.96121200
H	4.72203700	1.15639500	0.98333100
H	3.87499800	-0.07844300	1.90356200
H	2.84872000	2.77577000	1.31678500
H	1.89370900	1.55422900	2.17212400
C	3.52285900	2.33937400	3.26732300
O	2.87050000	2.65972800	4.24443200
O	4.86332600	2.39185500	3.24503900
H	5.17503600	2.71901200	4.11416100
C	3.71767400	-0.73085600	-0.84488000
C	4.86013300	-1.42441600	-0.39263900
C	3.17887400	-1.04097900	-2.11250000
C	5.44342500	-2.40925400	-1.19144800
H	5.29777200	-1.20131500	0.58149700
C	3.76740200	-2.02257900	-2.90697400
H	2.29852600	-0.49886800	-2.46279400
C	4.89977200	-2.70964000	-2.44662300
H	6.32659400	-2.94401100	-0.83461400
H	3.34668500	-2.25534500	-3.88797300
H	5.36037100	-3.48077000	-3.06926700

Table S2. Characteristics of selected NOCV pairs of **TS1-R**.

NOCV pair	ΔE kcal/mol	Eigenvalue
1	-89.30	1.16904
2	-27.50	0.50172
3	-11.29	0.38408
4	-13.02	0.33871
5	-13.65	0.29421
Overall ΔE_{orb} = -191.81 kcal/mol		

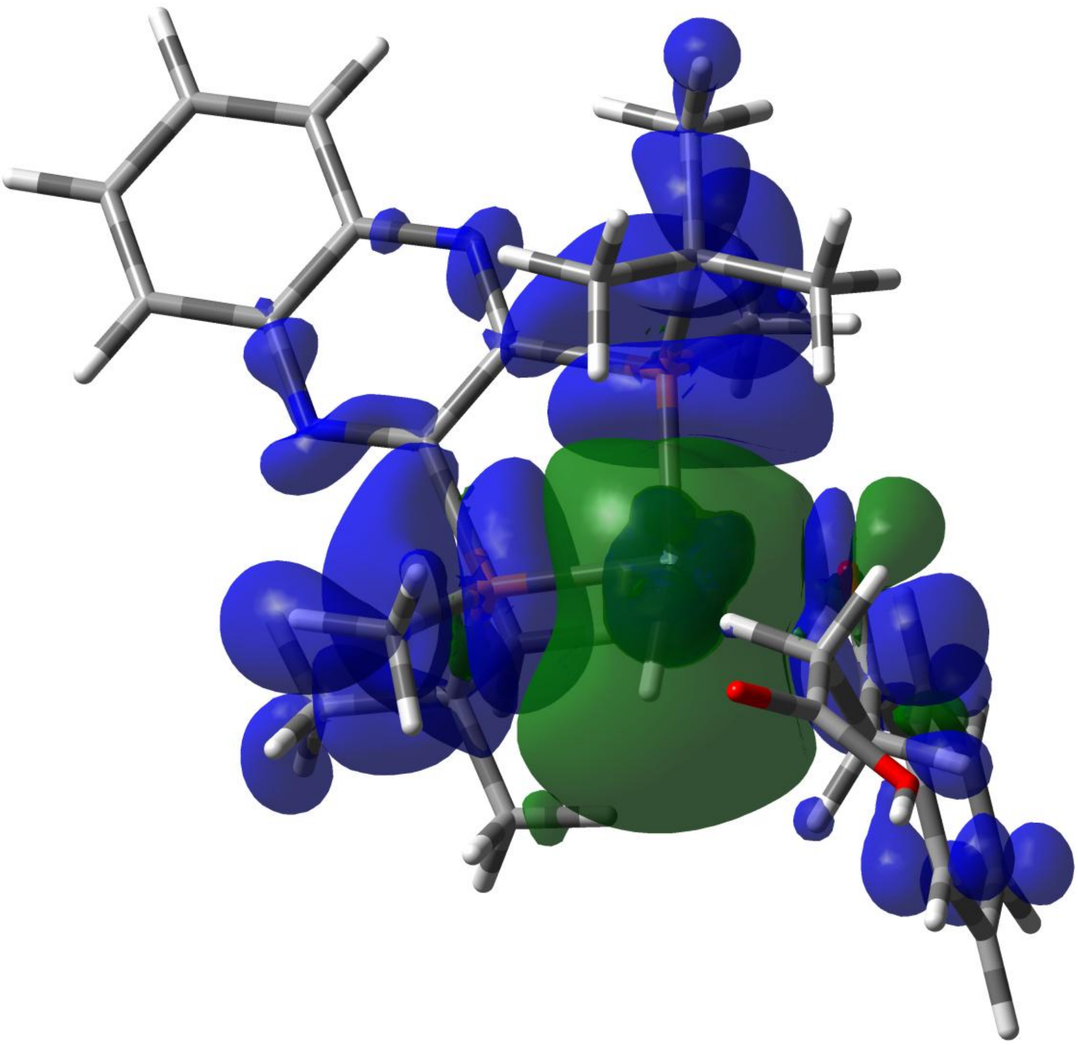

Figure S7. Visualization of sum of first and second NOCV pairs density isosurface for **TS1-R**. Isovalue = 0.004.

TS1-S (Cartesian coordinates for ETS-NOCV)

Charge 1; multiplicity 1

Ni	0.55894500	0.14757300	0.21170700
H	1.36397400	-0.64913600	1.19417200
P	-0.82997700	-1.13427100	1.14699500
P	-1.01776000	1.11445800	-1.03785000
C	-2.51741200	-0.70410900	0.49352900
C	-2.58336100	0.23305700	-0.58910700
N	-3.59894400	-1.27475400	0.98661400
N	-3.71912400	0.48723500	-1.21222700
C	-4.79074500	-0.99282600	0.39104000
C	-5.98733500	-1.57757900	0.88426300
H	-5.92879700	-2.22748800	1.75979600
C	-4.84485200	-0.12692400	-0.75426300
C	-6.09073900	0.10835400	-1.39452600
C	-7.18532900	-1.32269400	0.24653000
C	-7.23641800	-0.48160700	-0.89850100
H	-6.11257600	0.76332800	-2.26798200
H	-8.19737500	-0.30067200	-1.38590300
H	-8.10786500	-1.77342000	0.61988200
C	-0.60307900	-2.97256800	0.74329600
C	-1.63071900	-3.83012500	1.50207200
C	-0.77805300	-3.13709600	-0.77620300
C	0.81750500	-3.39094800	1.15892100
H	-1.47267000	-3.78768200	2.59082600
H	-2.66534000	-3.52729000	1.28715000
H	-1.50695200	-4.88047700	1.18770400
H	-0.07460300	-2.49859800	-1.33571600
H	-0.57566600	-4.18574500	-1.05162700
H	-1.80258200	-2.89664100	-1.10255300
H	0.92478100	-4.47654400	0.99422400
H	1.58760600	-2.87834500	0.56374100
H	1.01018600	-3.19349400	2.22560800
C	-1.01306700	-0.98985000	2.96121500
H	-1.93279700	-1.49431500	3.29103000
H	-0.13691600	-1.43719100	3.45296000
H	-1.06638700	0.07704000	3.22352500
C	-1.35919200	2.94221900	-0.66502200
C	-2.50014700	3.50905000	-1.52235900
C	-1.70487500	3.04231500	0.83071400
C	-0.05847600	3.71280300	-0.95658100
H	-2.25017000	3.49851600	-2.59465200
H	-3.44021600	2.95723400	-1.37896600
H	-2.67045800	4.56017000	-1.23129200
H	-0.90389600	2.61957100	1.45981200
H	-1.82730900	4.10360900	1.10596600
H	-2.64650000	2.52278200	1.07160400
H	-0.22815200	4.78432100	-0.75531700
H	0.77085600	3.37251000	-0.31938900

H	0.24983900	3.61604900	-2.00982300
C	-0.93255500	0.95022300	-2.86248500
H	-1.87651000	1.26303200	-3.33232600
H	-0.10223700	1.56453600	-3.24170000
H	-0.73395600	-0.10403200	-3.10706100
C	3.09800100	0.56441300	0.02412600
C	3.87146800	1.17044600	1.12627900
C	4.99570800	0.52768600	1.68781300
C	3.48370900	2.43637200	1.61809900
C	5.71242900	1.14007700	2.71692400
H	5.31049200	-0.45347700	1.32941400
C	4.20372900	3.04354000	2.64378900
H	2.61628800	2.93363100	1.18100700
C	5.31866100	2.39559600	3.19566900
H	6.57970900	0.63577600	3.14893000
H	3.89991900	4.02408700	3.01748300
H	5.88154700	2.87198700	4.00229900
C	3.58484200	-0.67583500	-0.68710800
H	2.71601500	-1.14132800	-1.17226900
H	4.01528200	-1.40068500	0.01653200
O	2.09218300	1.17278100	-0.44205300
C	4.62381000	-0.31155200	-1.75843200
H	5.52318100	0.15150800	-1.32078900
H	4.21533000	0.41950700	-2.47554000
C	5.06103400	-1.52820800	-2.53820900
O	4.64931400	-2.65973200	-2.36141800
O	5.97198900	-1.21952200	-3.47113900
H	6.21786100	-2.03858000	-3.94861700

Table S3. Characteristics of selected NOCV pairs of **TS1-S**.

NOCV pair	ΔE kcal/mol	Eigenvalue
1	-88.20	1.15953
2	-27.74	0.51345
3	-11.41	0.38553
4	-12.36	0.33288
5	-14.16	0.29727
Overall ΔE_{orb} = -190.17 kcal/mol		

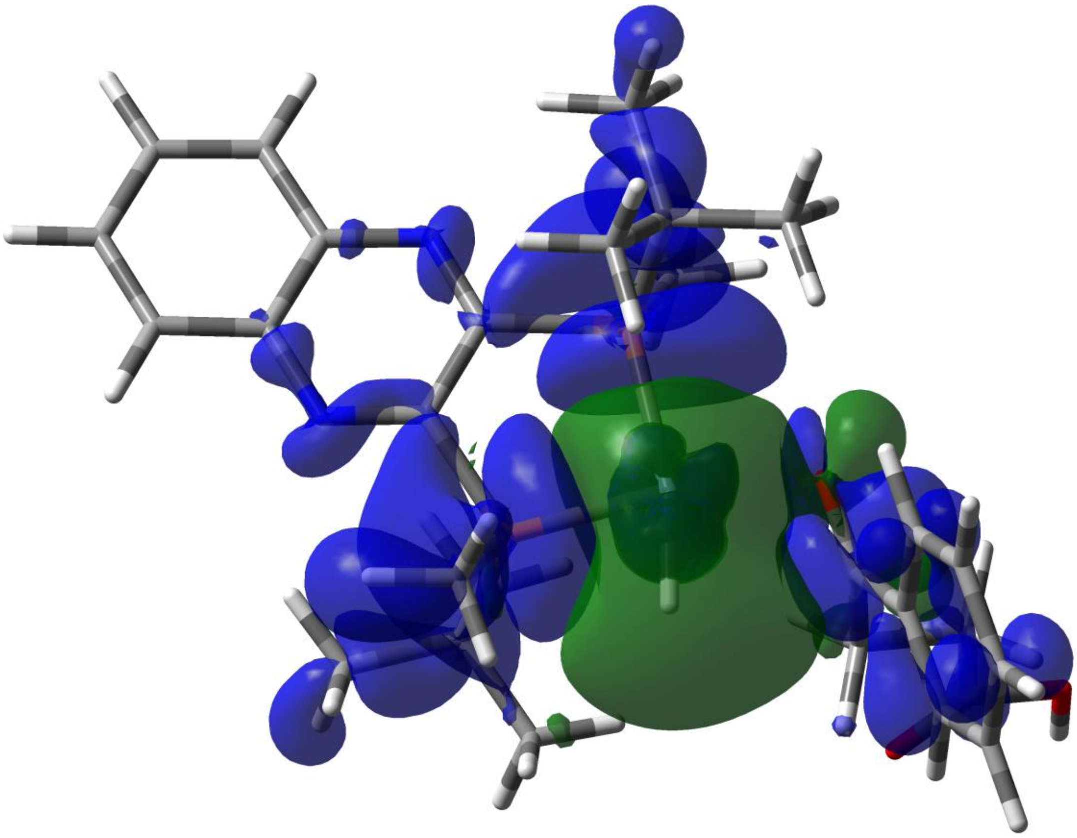

Figure S8. Visualization of sum of first and second NOCV pairs density isosurface for **TS1-S**. Isovalue = 0.004.
